# Age and *APOE* affect L-carnitine system metabolites in the brain in the APOE-TR model

**DOI:** 10.3389/fnagi.2022.1059017

**Published:** 2023-01-06

**Authors:** Claire J. C. Huguenard, Adam Cseresznye, Teresa Darcey, Aurore Nkiliza, James E. Evans, Stanley L. Hazen, Michael Mullan, Fiona Crawford, Laila Abdullah

**Affiliations:** ^1^Department of Metabolomics, Roskamp Institute, Sarasota, FL, United States; ^2^School of Life, Health and Chemical Sciences, Open University, Milton Keynes, United Kingdom; ^3^James A. Haley VA Hospital, Tampa, FL, United States; ^4^Department of Cardiovascular and Metabolic Sciences, Cleveland Clinic, Cleveland, OH, United States

**Keywords:** apolipoproteins, fatty acid metabolism, lipid oxidation, vascular biology, Alzheimer’s disease

## Abstract

With age the apolipoprotein E (*APOE*) E4 allele (involved in lipid homeostasis) is associated with perturbation of bioenergetics pathways in Alzheimer’s disease (AD). We therefore hypothesized that in aging mice *APOE* genotype would affect the L-carnitine system (central to lipid bioenergetics), in the brain and in the periphery. Using liquid chromatography-mass spectrometry, levels of L-carnitine and associated metabolites: γ-butyrobetaine (GBB), crotonobetaine, as well as acylcarnitines, were evaluated at 10-, 25-, and 50-weeks, in the brain and the periphery, in a targeted replacement mouse model of human *APOE* (APOE-TR). Aged APOE-TR mice were also orally administered 125 mg/kg of L-carnitine daily for 7 days followed by evaluation of brain, liver, and plasma L-carnitine system metabolites. Compared to E4-TR, an age-dependent increase among E2- and E3-TR mice was detected for medium- and long-chain acylcarnitines (MCA and LCA, respectively) within the cerebrovasculature and brain parenchyma. While following L-carnitine oral challenge, E4-TR mice had higher increases in the L-carnitine metabolites, GBB and crotonobetaine in the brain and a reduction of plasma to brain total acylcarnitine ratios compared to other genotypes. These studies suggest that with aging, the presence of the E4 allele may contribute to alterations in the L-carnitine bioenergetic system and to the generation of L-carnitine metabolites that could have detrimental effects on the vascular system. Collectively the E4 allele and aging may therefore contribute to AD pathogenesis through aging-related lipid bioenergetics as well as cerebrovascular dysfunctions.

## Introduction

The E4 allele of the *APOE* gene is one of the greatest genetic risk factor of late onset AD (Masters et al., 2015). It is also independently associated with abnormal lipid metabolism as well as an increased risk of cardiovascular and cerebrovascular disease compared to E3 and E2 alleles (Belloy et al., 2019; Yassine and Finch, 2020). The cerebrovascular system itself also plays a critical role in allowing the transport of nutrients, such as glucose and fatty acids (FA), from the periphery to the brain, for supporting neuronal and glial energetic needs. Among these, FAs and their intracellular transport into the mitochondria is important for their use as fuel (Longo et al., 2016), which may become particularly important in the brain when glucose supply is reduced (Yao et al., 2011).

The direct influence of the E4 allele on FA-related bioenergetics (i.e., the transport and use of metabolites, in this case FAs, for energy) within the cerebrovasculature requires further attention, specifically in E4 carriers because of their increased cerebrovascular vulnerability observed with age which could impair nutrient transport to the brain and affect proper neuronal and glial functioning (Ojo et al., 2021). A recent proteomic study suggested altered mitochondrial function in the cerebrovasculature isolated from deceased cognitively healthy E4 carriers compared to non-E4 carriers, indicating that cerebrovascular bioenergetics may indeed be altered in cognitively healthy E4 carriers (Ojo et al., 2021). While the role of the cerebrovascular system in regulating nutrient transport to the brain and the impairments in glucose transport in E4s has been shown in both clinical populations and animal models (Reiman et al., 2005; Alata et al., 2015; Zhao et al., 2017; Johnson et al., 2019), limited information is available regarding bioenergetics within the cerebrovasculature, particularly in relation to both FA uptake and fatty acid oxidation (FAO) capabilities within the context of aging and different *APOE* genotypes, despite the fact that cerebrovascular FAO has been suggested to be an essential process in maintaining its transport functions (Goldstein, 1979).

**Figure 1:**
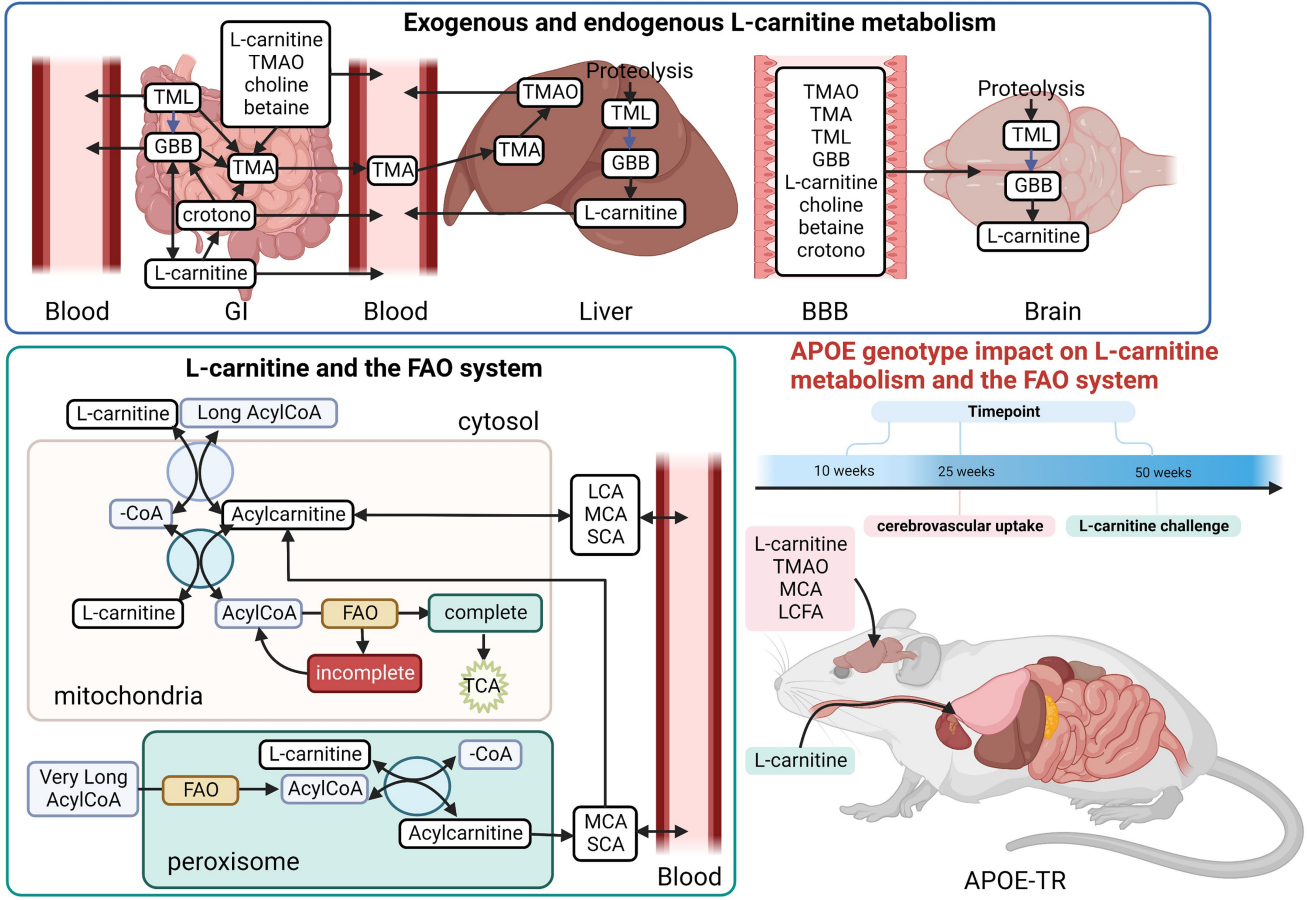
Overview of the L-carnitine system. Diagram showing an overview of the studies undertaken in the APOE-TR model. In the gut TMA-containing compounds obtained from the diet can be both directly absorbed into the blood and/or metabolized by the gut microbiome to yield TMA. Once in the bloodstream these metabolites enter tissues such as the liver and the brain. In the liver TMA is further oxidized to TMAO. Certain TMA containing compounds (TML, GBB, and L-carnitine) can also be biosynthesized by the liver and brain. Blue arrows in the L-carnitine biosynthesis pathways indicate some steps not shown. The exogenous and endogenous supplies of L-carnitine are important to the FAO pathway as activated long chain fatty acids (Long AcylCoA) can only enter the mitochondria through the L-carnitine shuttle system to undergo FAO. When there is an excess of intramitochondrial acyl-groups (during incomplete FAO) these can be exported out as acylcarnitines. The peroxisome also produces acylcarnitines from the chain shortening of activated very long chain fatty acids (Very Long AcylCoA). Here, we investigated the effect of *APOE* genotype and age on L-carnitine metabolite levels in the brain and the periphery. We also examined the effect of *APOE* on the cerebrovascular uptake of FAO-related compounds. Lastly, we interrogated the effect of *APOE* on the metabolism of exogenously supplied L-carnitine. BBB, blood brain barrier; crotono, crotonobetaine; FAO, fatty acid oxidation; GBB, γ-butyrobetaine; GI, gastro-intestinal tract; LCA, long chain acylcarnitines; LCFA, long chain fatty acids; MCA, medium chain acylcarnitines; SCA, short chain acylcarnitines; TMA, trimethylamine; TMAO, trimethylamine-n-oxide; TML, trimethyl-l-lysine; -CoA, free CoA. Made using Biorender.

The L-carnitine shuttle system is indispensable to FAO given that activated long chain fatty acids (LCFAs) have to be esterified with L-carnitine to form acylcarnitines in order to enter mitochondria (Ramsay et al., 2001; Figure 1). L-carnitine itself, as well as several of its metabolites, including GBB, crotonobetaine, and trimethylamine-n-oxide (TMAO), have been associated with cerebrovascular dysfunction and cardiovascular disease (Koeth et al., 2013, 2014, 2019; Luciani et al., 2021). L-carnitine and other trimethylamine (TMA) containing compounds (including GBB and crotonobetaine) can be further metabolized by gut bacteria to yield free TMA (Koeth et al., 2014; Figure 1), which is then absorbed into the bloodstream and converted to TMAO in the liver (Wang et al., 2011). Since there are known *APOE*-dependent differences in the gut microbiota in both humans and APOE-TR mice (Tran et al., 2019), L-carnitine metabolism could be impacted and, in turn, affect cerebrovascular function by disturbing normal L-carnitine shuttle function, particularly if other vulnerabilities are present. However, the effects of different *APOE* genotypes on the L-carnitine system, upon which FAO is largely dependent, have not yet been examined either.

Given that the E4 allele is associated with both an increased reliance on alternative fuels, such as FAs, and more severe cerebrovascular dysfunction with age (Yassine and Finch, 2020; Ojo et al., 2021), it is possible that, aging E4 carriers may display alterations in L-carnitine, L-carnitine gut metabolites, and acylcarnitine profiles in the brain and the periphery reflecting such alterations. We therefore hypothesized that with age different *APOE* genotypes would be associated with changes in L-carnitine, L-carnitine metabolites and acylcarnitines in the brain and the periphery. To address this, we analyzed these metabolites in isolated cerebral vessels and in the brain parenchyma over a range of ages in the APOE-TR mouse model. An *ex vivo* study of FA uptake and metabolism was also conducted in isolated cerebral vessels from APOE-TR mice. Additionally, L-carnitine was orally administered to APOE-TR mice and its metabolism in the periphery and the brain evaluated (Figure 1). As multiple studies now show an impact of *APOE* on brain bioenergetics, which occurs prior to the onset of significant AD pathologies (Reiman et al., 1996), it is anticipated that improving our understanding of the L-carnitine system will help us better appreciate these early bioenergetics changes that could precede brain amyloid and tau pathologies and as such provide avenues for AD prevention.

## Materials and methods

### Animal models

In these studies, APOE-TR mice were housed in groups of up to four under standard laboratory conditions at 23 ± 1°C, 50 ± 5% humidity, with a 12-h light/dark cycle and access to food and water *ad libitum*. The APOE-TR model is a mouse knock-in model where exons 2–4 (comprising the mouse *APOE* gene) are replaced with human *APOE* in 129xC57BL/6 mice back-crossed to C57BL/6 mice (Sullivan et al., 1997). All procedures were carried out under Institutional Animal Care and Use Committee approval and in accordance with the National Institutes of Health Guide for the Care and Use of Laboratory Animals. Mice were humanely euthanized by cardiac puncture after being anesthetized in an anesthetic chamber with 3% isoflurane at a 2.5 L/min (min) O_2_ flow rate.

For the timepoint study (*n* = 50), male and female APOE-TR mice were euthanized at three different timepoint (10-, 25-, and 50-weeks) for three homozygous genotypes: APOE2-TR, APOE3-TR, and APOE4-TR (Supplementary Table S1). For the cerebrovascular uptake study (*n* = 22), male and female homozygous APOE2-, APOE3-, and APOE4-TR mice were euthanized at 26-weeks (Supplementary Table S2). For the L-carnitine challenge study (*n* = 47), male and female homozygous APOE2-, APOE3-, and APOE4-TR mice were euthanized at 53-weeks (Supplementary Table S3).

### Blood and tissue processing

For all studies blood was drawn by cardiac puncture in anesthetized mice and collected in 5 μl EDTA (BD medical) before being spun in a microcentrifuge at 9.8 revolutions per minute (RPM) for 5 min at room temperature. The plasma supernatant was collected immediately and placed in liquid nitrogen. For the timepoint study, the whole liver was dissected out and immediately placed in liquid nitrogen. Each liver was then homogenized in a dounce homogenizer (Cole Parmer) with 2 ml of lysis buffer. The lysis buffer was prepared by mixing 10 ml of M-PER (ThermoFisher) with 100 μl of Protease & Phosphatase inhibitor mix (ThermoScientific) and 100 μl of 0.5 M EDTA (ThermoScientific). The liver homogenates were then transferred to dry ice and stored at −80°C. The whole brain was also dissected out and immediately transferred to dry-ice and kept at −80°C until further processing. For the brain fractionation as per Eisenbaum et al. (2021), the brain was homogenized in 3 ml of Hank’s balanced salt solution (HBSS, Millipore Sigma) using a dounce homogenizer (Cole Parmer). Then 125 μl of brain homogenate were collected with 125 μl of lysis buffer (made as previously described). Following this, 5 ml of 40% dextran solution were added to the rest of the homogenate before being centrifuged at 6000 RCF and 4°C for 15 min using a fixed angle rotor. This procedure resulted in a pellet containing the cerebrovasculature and a floating parenchymal layer separated by a dextran interface. The capillary pellet was collected with 250 μl of lysis buffer and the brain parenchymal fraction was collected with 250 μl of lysis buffer. Samples were stored at −80°C until the day of analysis. For the cerebrovascular uptake studies, cerebrovascular fractions were extracted from fresh brains and for each brain the cerebrovascular fraction was divided into two aliquots one used for vehicle treatment and the other for compound treatment. These two aliquots were incubated at 37°C for 20 min with their respective treatment solutions. After incubation the cerebrovascular fractions were washed three times with PBS and collected in 500 μl of lysis buffer.

The Thermo Scientific™ Pierce™ bicinchoninic acid Protein Assay Kit was used to quantify proteins in the tissue homogenates analyzed. Samples were run in duplicates on 96-well plates in a randomized manner with a cut-off CV of 15%.

### Preparation of treatment and vehicle solutions for cerebrovascular uptake

Solutions were made fresh from the same stocks on the day of each treatment. A stock solution containing; 0.001 M TMAO (Fisher Scientific), 0.002 M L-Carnitine (Ambeed), 2 μM C6:0-CAR (Cayman Chemicals) and 2 μM C12:0-CAR (Cayman Chemicals) was prepared in methanol (MeOH) and stored at −80°C until the day of the experiment. Both uniformly C13-labeled palmitic acid (UC13-C16:0, Larodan) and docosahexaenoic acid (DHA, Cayman Chemicals) were conjugated with albumin (using Fatty Acid Free Bovine Serum Albumin, BSA, Goldbio) to a final concentration of 2 mM and 40 μM, respectively, and aliquoted out into 5 ml glass scintillation vials stored at −20°C. The acylcarnitine mix solution and the Fatty acid-BSA conjugate were combined to make the final treatment solution with a final concentration of 5 μM TMAO, 10 μM L-carnitine, 10 nM C6:0-CAR, 10 nM C12:0-CAR, 1 mM UC13-C16:0 and 20 μM DHA. The vehicle solution was prepared in the same way without the addition of compounds and contained the same Fatty Acid Free BSA. L-carnitine, GBB, TMAO, acylcarnitines, UC13-C16:0, and DHA were measured in the BSA vehicle solution to determine their background level. As expected, since a fatty acid free BSA solution was used, the analytes detected represented ≤1% of the signals detected in the cerebrovasculature.

### Cytotoxicity assay

The CyQUANT™ lactate dehydrogenase (LDH) Cytotoxicity Assay (Invitrogen™) was used to examine treatment-related cytotoxicity. The cerebrovasculature was extracted and treated as above. After being collected in 500 μl of PBS the capillary fraction was divided into three aliquots, one for the maximum LDH release, one for the vehicle treatment induced LDH release (spontaneous LDH release) and one for the treatment induced LDH release. For the maximum LDH release the capillary fraction was resuspended 160 μl PBS with 16 μl of 10× lysis buffer and incubated at 37°C for 45 min as per the manufacturer’s instruction. The supernatant was then collected and put on ice until measurement. The vehicle and treated fractions tubes were resuspended in the vehicle and treated solution each and incubated at 37°C for 20 min. The supernatants were collected and put on ice until measurement. When all samples were ready 50 μl of each were plated in duplicate into a 96-well plate along with a positive control. A 15% CV cut-off was used. The % Cytotoxicity was calculated using the following formula:


$$\%Cytotoxicity=\left[\frac{\begin{array}{l} Compoundtreated\;LDH\;activity\\ -Spontaneous\;LDH\;activity \end{array}}{\begin{array}{l} Maximum\;LDH\;activity\\ -Spontaneous\;LDH\;acitivity \end{array}}\right]\times 100$$

### L-carnitine oral challenge

Seven weeks before the start of the study, mice were housed in groups of four according to sex, *APOE* genotype, and treatment group (Supplementary Table S3); bedding was exchanged between all cages twice a week for the first 2 weeks in order to normalize for microbiome differences between cages. Then bedding was exchanged only between mice of the same genotypes twice a week for the remaining 5 weeks before the beginning of treatment [due to the known differences in microbiome between APOE-TR mice (Tran et al., 2019)]. Mice were orally gavaged every day for 7 days with either PBS vehicle (Thermo Fisher Scientific) or L-carnitine (Sigma Aldrich) at a dose of 125 mg/kg. This dose and time regiment was chosen as has been shown to be well-tolerated in mice while producing measurable biological effects on the mitochondria (Makowski et al., 2009; Xia et al., 2011). During gavage mice were under light anesthesia to limit discomfort (3% isoflurane at a 1.5 L/min O_2_ flow rate for ~1 min). Each day mice were kept on a heated blanket after gavage and observed for at least 10 min. After being returned to their racks mice were checked for signs of pain and distress and their weight monitored daily. Mice were euthanized ~24 h after the last gavage.

### AST ELISA

A mouse Aspartate Aminotransferase (AST) SimpleStep ELISA kit (abcam, ab263882) was used to measure AST in mouse plasma. The plasma samples were diluted 100× and the assay conducted as per the manufacturer’s instruction. Samples were run in duplicate in a randomized manner on 96-well plates. The plates were read at 450 nm and concentrations obtained in pg./ml from a standard curve using a 4-parameter curve fit with blank control subtracted absorbance values and a 15% CV cut-off. The dynamic range of the assay was 125–8,000 pg/ml.

### Acylcarnitine assay

Acylcarnitines were extracted from either 10 μl of whole liver homogenate, 50 μl of plasma, 50 μl brain parenchyma, 50 μl cerebrovasculature (timepoint study), 60 μl of cerebrovasculature (cerebrovascular uptake study) or 100 μl of brain homogenate (all homogenized as described above) and spiked with 5 μl of acylcarnitine internal standard (IS) mix containing 12 μM TMAO-d9 (Cambridge Isotope Laboratories, Inc., CIL), NSK-B (CIL) and NSK-B-G1 (CIL) at the beginning of the extraction process. Protein precipitation was performed by adding 10 volumes of 25% MeOH in acetonitrile (ACN), except for the brain homogenate which was extracted in 100% MeOH. This was followed by vortex mixing for ~1 min before centrifuging samples at 10,000 relative centrifugal force (RCF) for 20 min at 4°C. Then 80% of the supernatant was taken and dried down before reconstitution in 100 μl of mobile phase A (90:5:5 ACN:H_2_O:100 mM ammonium formate (AmFm) in H_2_O) and vortexed for ~10 s. Samples were then transferred to 0.2 μm centrifugal filters and centrifuged again at 10,000 RCF for 5 min at 4°C before being transferred to glass auto-sampler vials ready for injection.

A Shimadzu Prominence Ultra-Fast Liquid Chromatographer (LC) interfaced to a Thermo Scientific Q Exactive Mass Spectrometer (MS) with a Thermo Scientific™ heated electrospray ionization (HESI-II) probe was used for LC–MS/MS. Chromatography was performed on a Kinetex 2.6 μm HILIC 100 Å, 100 × 2.1 mm internal diameter column (Phenomenex), with a constant flow rate of 250 μl/min and a solvent gradient from 20% Mobile Phase B (50:45:5, ACN:H_2_O:100 mM AmFm in H_2_O) to 22% B at 5 min, 40% B at 10 min, 60% B at 13 min, 80% B at 15 min, and 99% B at 20 min. The column was re-equilibrated at 20% B for 5 min before the next sample injection. All samples were kept at 5°C in the auto-sampler tray for the duration of the analysis. Data was acquired in positive mode using parallel reaction monitoring with an inclusion list of acylcarnitines of interest (see Supplementary Table S4). Acylcarnitine species were abbreviated as follows: Cx:y-CAR, acylcarnitine; Cx:y-OH-CAR, hydroxy acylcarnitine; Cx:y-DC-CAR, dicarboxy acylcarnitine.

### TMA-containing compound assay

Trimethylamine-containing compounds were extracted from 50 μl of plasma, 50 μl of whole liver homogenate or from 100 μl of whole brain homogenate (without the cerebellum and olfactory bulbs) by adding 10 volumes of ACN and spiking the samples with 5 μl of IS mix containing 12 μM d9-TMAO (CIL), 649 nM d9-TMA (CDN Isotopes), 6 μM d3-L-carnitine (CIL), 329 nM d9-crotonobetaine (see Koeth et al., 2014) and 5 μM d9-TML (Santa Cruz Biotechnology) at the beginning of the extraction. Samples were vortexed and left on ice for 30 min before being centrifuged at 10,500 revolutions per minute (RPM) at 4°C for 5 min. Then 80% of the supernatant was transferred to a new tube and 2 μl of concentrated ammonia solution added, followed by 100 μl of 4 mg/ml ethyl bromoacetate (Oakwood chemicals) in ACN and samples vortex mixed. The samples were left to react for 10 min before the solution was quenched with 10 μl of formic acid. The samples were then vortexed and dried down using a nitrogen evaporator. After this 250 μl MeOH were added, and the samples were vortexed and centrifuged at 10,500 RPM at 4°C for 5 min. Then 80% of the supernatant was transferred to 0.2 μm centrifugal filter tubes and samples were centrifuged again at 10,500 RPM at 4°C for 5 min. Samples were dried down again in a nitrogen evaporator and reconstituted in 100 μl 80% of ACN + 2% formic acid. Finally, the extracts were vortexed and transferred to glass auto-sampler vials for further analysis.

An LTQ Orbitrap MS interfaced to an Agilent 1200 LC was used for LC–MS/MS analysis. Samples were separated using a Raptor HILIC-Si 2.1 × 100 mm, 2.7 μm LC column (Restek) with a constant flow rate of 400 μl/min following a flow gradient starting at 5% Mobile Phase B (H_2_O + 5 mM AmFm +0.5% formic acid) then going up to 40% in 5 min, which was followed by re-equilibration at 5% B for 4 min and 30s. Mobile phase A was 90% ACN + 5 mM AmFm +0.5% formic acid. All samples were kept at 5°C in the auto-sampler tray for the duration of the analysis. Data was acquired in positive mode using full scan with a mass range of *m/z* 75–200 and resolution of 30,000. For increased sensitivity, data was also acquired using positive mode with single ion monitoring for TML and d9-TML, *m/z* 189 ± 4 and 198.22 ± 4, respectively, at a resolution of 7,500. Using this assay, a list of trimethylamine-containing compounds were measured (see Supplementary Table S5).

### Palmitic acid-DHA assay

The fatty acids UC13-C16:0 and DHA were extracted from 40 μl of cerebrovasculature by adding 10 volumes of isopropanol (IPA) and spiking samples with 5 μl of 12 μM d2-C16:0 (Cayman Chemicals) and 0.67 μM d5-DHA (Cayman Chemicals) at the beginning of the extraction. Samples were vortexed and centrifuged at 10,000 RPM at 4°C for 5 min. Then 80% of the supernatant was transferred to a different tube and dried down using a nitrogen evaporator. Samples were then reconstituted in 45 μl 50% ACN and transferred to glass autosampler vials for injection.

An LTQ Orbitrap MS interfaced to an Agilent 1200 LC was used for LC–MS analysis. Samples were separated using a Kinetex^®^ 2.6 μm XB-C18 100 Å, 100 × 2.1 mm LC Column (Phenomenex) with a constant flow rate of 250 μl/min following a flow gradient starting at 70% Mobile Phase B (ACN + 0.5% Acetic acid, AA) then going up to 99% for 5 min before re-equilibration at 70% for 5 min. Mobile phase A was H_2_O + 0.5% AA. All samples were kept at 5°C in in the auto-sampler tray for the duration of the analysis. For DHA and d5-DHA data were acquired in negative mode using full scan with a mass range of *m/z* 326–335 at a resolution of 30,000. The UC13-C16:0 and d2-C16:0 data were also acquired in negative mode using full scan with a mass range of *m/z* 256–273 at resolution of 30,000. Using this assay UC13-C16:0 and DHA were measured (see Supplementary Table S6).

### Data processing and statistical analysis

Peak areas were obtained from Tracefinder™ with a target compound list of compounds of interests at a window of five parts per million accuracy (see Supplementary Tables S4–S6). Concentrations were calculated based on relative spiked IS amount, with data normalized across runs using the quality control sample that was run along with each batch. Each sample was injected in triplicate, and all triplicate runs with a CV above 20% were excluded from further analysis. After concentration calculation and data cleanup, data was uploaded to SPSS for statistical analysis.

Normal distribution of the data was assessed by examining mean skewness and kurtosis for each group with values −2≤ or ≥2 considered reflective of non-normal distribution. Non-normally distributed data was transformed using the natural log function (ln) and tested for normality again. Data that could not be normalized using the ln were analyzed using non-parametric tests. For normally distributed data a mixed linear model (MLM) was used, (as laid out by IBM in their SPSS Statistics guidelines, https://www.ibm.com/docs/en/spss-statistics/29.0.0?topic=statistics-linear-mixed-models), with mice IDs as subject variable and random effects, sex and *APOE* genotypes as factors, and age as a covariate. Additionally, for the cerebrovascular uptake study treatment was added as repeated variable. Both main effects and two variable interactions were analyzed. When comparing more than 2 groups, if the F tests of fixed effects was statistically significant (*p* < 0.05) a pair-wise comparisons with least significant difference (LSD) were performed with correction for multiple comparisons using the Benjamini-Hochberg (B-H) procedure as described in (Huguenard et al., 2020). For non-normally distributed data independent-samples non-parametric tests were used (either Mann–Whitney *U*-test for 2 group analysis or the Kruskal-Wallis 1-way ANOVA for more than 2 groups with B-H correction) or non-parametric related samples analysis for the cerebrovascular uptake study. For correlation analyses Kendall’s tau-b was used with two tailed-test and a *p* threshold of *p* < 0.05. For Short Chain Acylcarnitines (SCAs) calculation, acylcarnitines of chain lengths C2-C5 were summed, for MCAs chain lengths C6-C12 were summed, for LCAs chain lengths C13-C21 were summed, and for very long chain acylcarnitines (VLCAs) chain lengths ≥ C22 were summed. For total acylcarnitines, all chain lengths were summed. These composite variables excluded: L-carnitine, its TMA-containing related metabolites, hydroxyl- and dicarboxyl-species.

Graphs were built using the GraphPad Prism 9.2.0 software and MetaboAnalyst 5.0.^1^ The diagram figure was drawn using BioRender.^2^

### Study design

To briefly summarize the study strategy, we firstly investigated the effects of *APOE* and age (while also considering sex) first on individual acylcarnitine species, then on chain length groups (as acylcarnitines of similar chain length are highly correlated), and total acylcarnitines (which can reveal broader changes affecting acylcarnitines) in the brain (parenchyma and cerebrovasculature) and the periphery (liver and plasma). The second and third parts of our study investigated metabolism and transport of metabolites relevant to the L-carnitine system both centrally (in the cerebrovasculature) and peripherally. The effect of *APOE* on the cerebrovascular uptake of FAs, and other L-carnitine related metabolites was examined first and lastly differences in peripheral L-carnitine metabolism with different *APOE* genotypes was explored (see Figure 1 for a summary of studies and L-carnitine metabolic pathways).

## Results

### Age, *APOE*, and sex affect brain parenchymal and cerebrovascular acylcarnitines

**Figure 2:**
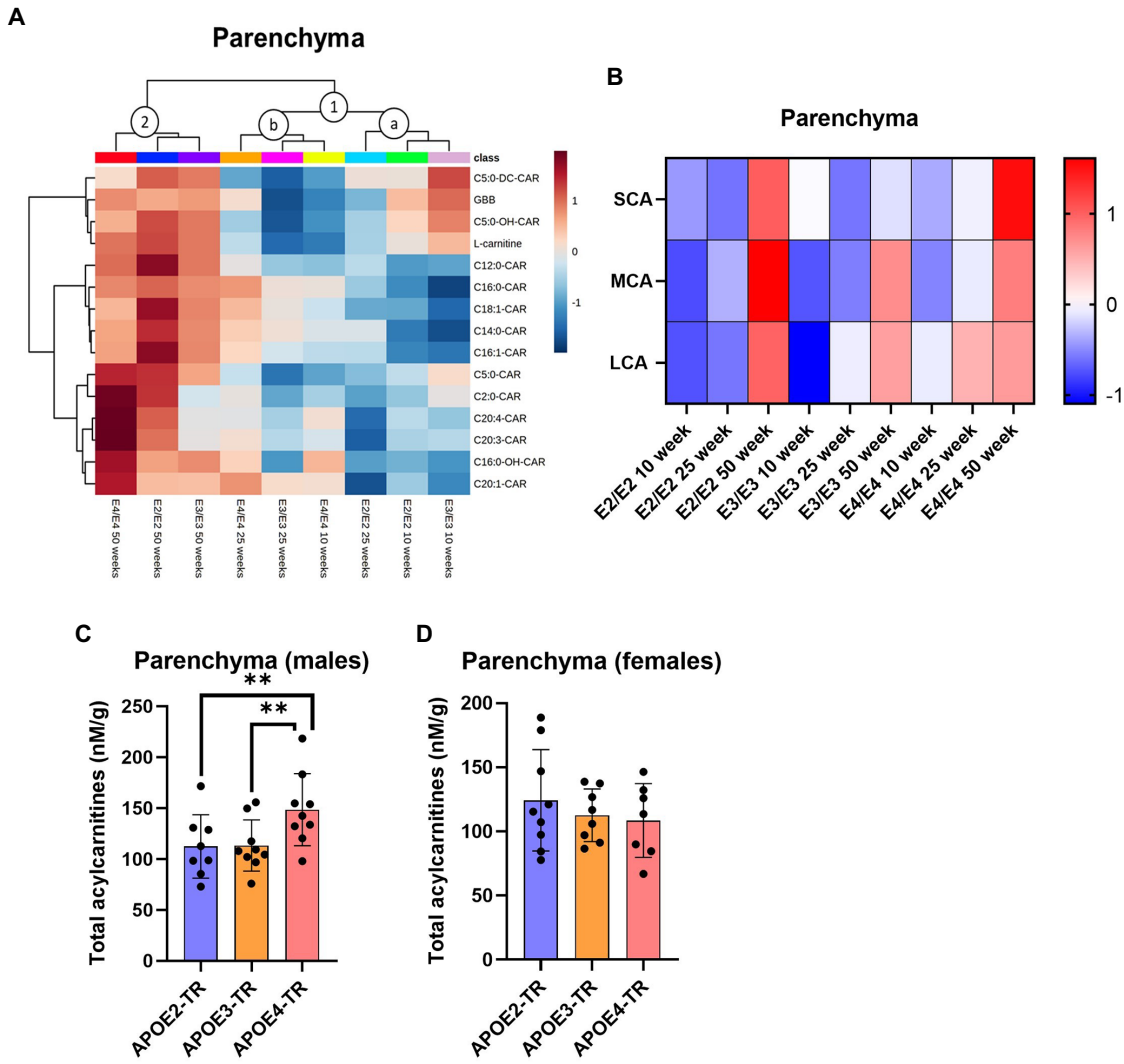
Age leads to increases in brain parenchymal acylcarnitines in an *APOE*-dependent manner. **(A)** Hierarchical clustering heatmap showing acylcarnitine species with *APOE* genotypes at different age timepoints in the brain parenchyma. **(B)** Z-score heatmap showing acylcarnitine chain length ratios to total acylcarnitines with *APOE* genotypes at different age timepoints in the brain parenchyma. **(C)** Bar graphs showing all points with mean ± SD total acylcarnitines in nM per gram of protein with *APOE* genotype in males in the brain parenchyma. **(D)** Bar graphs showing all points with mean ± SD total acylcarnitines in nM per gram of protein with *APOE* genotype in females in the brain parenchyma. Statistics: For the clustering heatmap data were normalized and scaled before being analyzed using Ward’s clustering, with the top 15 acylcarnitines selected by ANOVA. Multiple comparisons with B-H correction were performed on the data, *p < 0.05, **p < 0.01, ***p < 0.001. Numbers per group; 10-week APOE2-TR *n* = 6, 25-week APOE2-TR *n* = 5, 50-week APOE2-TR *n* = 6, 10-week APOE3-TR *n* = 6, 25-week APOE3-TR *n* = 5, 50-week APOE3-TR *n* = 6, 10-week APOE4-TR *n* = 6, 25-week APOE4-TR *n* = 6, 50-week APOE4-TR *n* = 4, APOE2-TR (males) *n* = 8, APOE3-TR (males) *n* = 9, APOE4-TR (males) *n* = 9, APOE2-TR (females) *n* = 9, APOE3-TR (females) *n* = 8, APOE4-TR (females) *n* = 7. Cx:y-CAR, acylcarnitines; Cx:y-OH-CAR, hydroxy acylcarnitines; Cx:y-DC-CAR, dicarboxy acylcarnitines; GBB, γ-butyrobetaine; LCA, long chain acylcarnitines; MCA, medium chain acylcarnitines; SCA, short chain acylcarnitines; TMAO, Trimethylamine-n-oxide.

In the brain parenchyma and the cerebrovasculature, there was an independent effect of age, which was further modulated by *APOE* genotype on L-carnitine system metabolites (Supplementary Figure S1). In the brain parenchyma, there was an age and APOE dependent clustering of acylcarnitine species (see clusters 1a, 1b, and 2, Figure 2A). In a first cluster several odd chain acylcarnitine (OCA) species, C5:0-DC, C5:0-OH, along with GBB and L-carnitine, were higher in 10-week-old E3 mice compared to 10-week-old E2 and E4 mice (clusters 1a and 1b, Figure 2A; Supplementary Figure S2A). A second major cluster included the 50-week-old age group which showed highest increases in acylcarnitines among all timepoints (cluster 2, Figure 2A; Supplementary Figure S2A). When examining differences between chain length groups, there was a significant effect of age on SCAs, the MCA C12:0-CAR, and LCAs (*p* ≤ 0.001), which all increased from 10 to 50 weeks (Figure 2B). There was also a main effect of *APOE* genotype on LCA levels (*p* = 0.018), which were marginally higher in E4s compared to E3s (*p* = 0.067; Figure 2B). Total acylcarnitine levels increased with age in E2 and E3 mice but not in E4 mice (Supplementary Figures S3A–D). Total acylcarnitines were also increased in male E4 mice compared to males of other genotypes, while the same sex-*APOE* genotype effects were not detected in females, with no significant age interactions (Figures 2C,D). Lastly, TMAO measured in whole brain homogenate was significantly elevated in E3 mice compared to other genotypes with no additional effects of age or sex (Supplementary Figure S3I).

**Figure 3:**
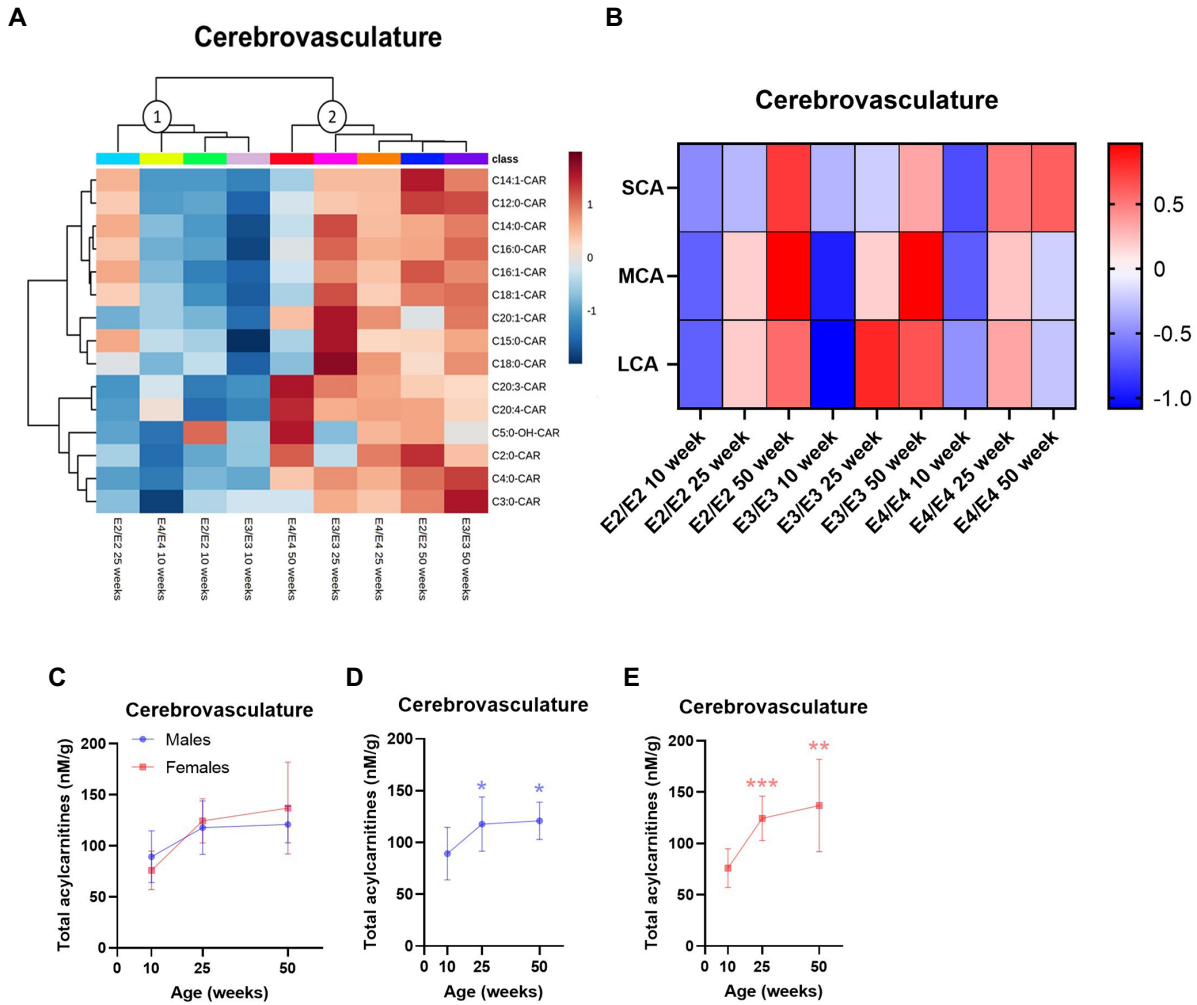
Age leads to increases in cerebrovascular acylcarnitines in an *APOE-*dependent manner. **(A)** Hierarchical clustering heatmap showing acylcarnitine species with *APOE* genotypes at different age timepoints in the cerebrovasculature. **(B)** Z-score heatmap showing acylcarnitine chain length ratios to total acylcarnitines with *APOE* genotypes at different age timepoints in the cerebrovasculature. **(C)** Line graphs showing mean ± SD total acylcarnitines in nM per gram of protein in males and females at different age timepoints in the cerebrovasculature. **(D)** Line graphs showing mean ± SD total acylcarnitines in nM per gram of protein in males at different age timepoints in the cerebrovasculature, * comparison with 10  weeks. **(E)** Line graphs showing mean ± SD total acylcarnitines in nM per gram of protein in females at different age timepoints in the cerebrovasculature, * comparison with 10 weeks. Statistics: For the clustering heatmap data were normalized and scaled before being analyzed using Ward’s clustering, with the top 15 acylcarnitines selected by ANOVA. Multiple comparisons with B-H correction were performed on the data, *p < 0.05, **p < 0.01, ***p < 0.001. Numbers per group; 10-week APOE2-TR *n* = 6, 25-week APOE2-TR *n* = 5, 50-week APOE2-TR *n* = 6, 10-week APOE3-TR *n* = 6, 25-week APOE3-TR *n* = 5, 50-week APOE3-TR *n* = 6, 10-week APOE4-TR *n* = 6, 25-week APOE4-TR *n* = 6, 50-week APOE4-TR *n* = 4, 10-week (males) *n* = 9, 25-week (males) *n* = 8, 50-week (males) *n* = 9, 10-week (females) *n* = 9, 25-week (females) *n* = 8, 50-week (females) *n* = 7. Cx:y-CAR, acylcarnitines; Cx:y-OH-CAR, hydroxy acylcarnitines; LCA, long chain acylcarnitines; MCA, medium chain acylcarnitines; SCA, short chain acylcarnitines.

In the cerebrovasculature, there was an increase in acylcarnitine species with age which was affected by *APOE* genotype. Acylcarnitine profiles in 25-week-old E2 mice were similar to that of younger (10-week-old) mice of other *APOE* genotypes (cluster 1, Figure 3A) and 25- and 50-week-old E3 and E4 mice had similar profiles (cluster 2, Figure 3A). When examining chain length groups in both E2 and E3 mice, there was an increase in several LCAs, the MCA C12:0-CAR as well as SCAs from 10 to 50 weeks (*p* ≤ 0.01), however this was not detected in E4 mice (Figure 3B; Supplementary Figure S2B). Accordingly, there was a significant overall increase in total acylcarnitines in E2 and E3 mice with age, but not in E4 mice (Supplementary Figures S3E–H). The increase in total acylcarnitine levels with age was also more significant in females than in males (Figures 3C–E).

### Sex and *APOE* effects dominate peripheral acylcarnitine profiles

**Figure 4:**
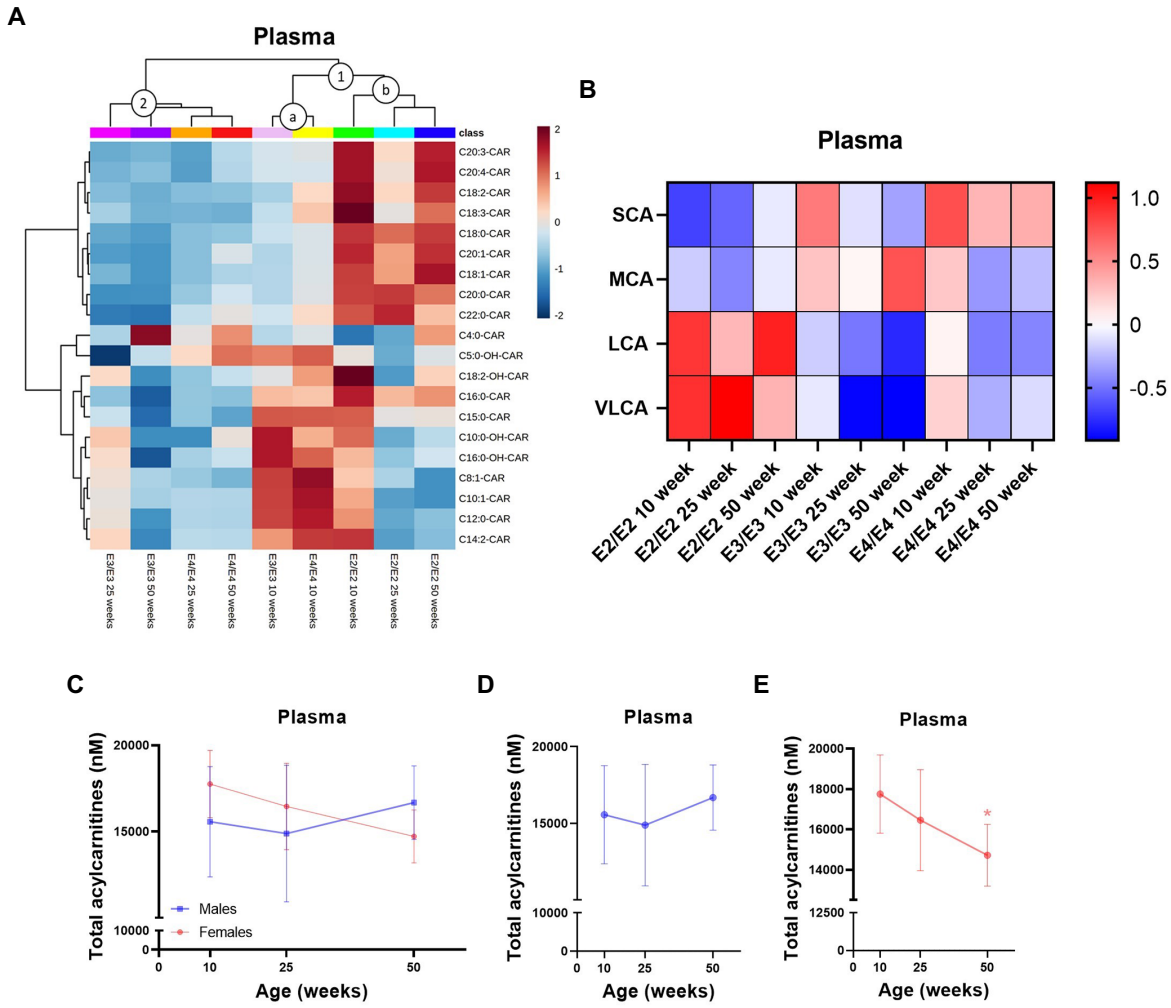
Plasma acylcarnitines are affected by *APOE*, age, and sex. **(A)** Hierarchical clustering heatmap showing acylcarnitine species with *APOE* genotypes at different age timepoints in plasma. **(B)** Z-score heatmap showing acylcarnitine chain length ratios to total acylcarnitines with *APOE* genotypes at different age timepoints in plasma. **(C)** Line graphs showing mean plasma total acylcarnitines ± SD in nM in males and females at different age timepoints. **(D)** Line graphs showing mean plasma total acylcarnitines ± SD in nM in males at different age timepoints. **(E)** Line graphs showing mean plasma total acylcarnitines ± SD in nM in females at different age timepoints, * comparison with 10 weeks. Statistics: For the clustering heatmap data were normalized and scaled before being analyzed using Ward’s clustering, with the top 20 acylcarnitines selected by ANOVA. Multiple comparisons with B-H correction were performed on the data, *p < 0.05, **p < 0.01, ***p < 0.001. Numbers per group; 10-week APOE2-TR *n* = 6, 25-week APOE2-TR *n* = 5, 50-week APOE2-TR *n* = 6, 10-week APOE3-TR *n* = 6, 25-week APOE3-TR *n* = 5, 50-week APOE3-TR *n* = 6, 10-week APOE4-TR *n* = 6, 25-week APOE4-TR *n* = 6, 50-week APOE4-TR *n* = 4, 10-week (males) *n* = 9, 25-week (males) *n* = 8, 50-week (males) *n* = 9, 10-week (females) *n* = 9, 25-week (females) *n* = 8, 50-week (females) *n* = 7. Cx:y-CAR, acylcarnitines; Cx:y-OH-CAR, hydroxy acylcarnitines; LCA, long chain acylcarnitines; MCA, medium chain acylcarnitines; SCA, short chain acylcarnitines; VLCA, very long chain acylcarnitines.

In plasma, *APOE* genotype, age, and sex all affected L-carnitine, its metabolites as well as acylcarnitine levels while in the liver there was a strong effect of sex which was further modulated by *APOE* genotype (Supplementary Figure S1). Of the two main clusters in plasma, (1 and 2, Figure 4A), one sub-cluster composed of 10-week-old E3 and E4 mice had higher MCAs but lower LCAs relative to other clusters (cluster 1a, Figure 4A), while the second sub-cluster contained all age groups of E2 mice with a reverse trend of higher LCAs with age (cluster 1b, Figure 4A). The second main cluster was composed of 25- and 50-week-old E3 and E4 mice which had lower levels of acylcarnitines across all chain lengths (cluster 2, Figure 4A). In E2 and E4 mice, the MCA C12:0-CAR was significantly lower at 25- and 50-week but only at 50 week of age in E3 mice (Supplementary Figure S4A). In plasma, several LCAs were lower with increasing age, while they remained higher in E2 mice (Supplementary Figure S4A). Regardless of age, SCAs were higher in E4 mice compared to other genotypes and LCAs and the C22:0-CAR VLCA were higher in E2 mice compared to other genotypes (Figure 4B; Supplementary Figures S5G,H). Levels of TMAO were significantly increased in females compared to males (Supplementary Figure S4A). Females also had higher levels of the MCA C12:0-CAR, and several LCAs compared to males (Supplementary Figure S4A). In females, there was also a significant decrease in plasma total acylcarnitines with age but not in males (Figures 4C–E). As with the brain, TMAO was significantly increased in male E3 mice compared to males from other genotypes with a similar trend for females (Supplementary Figures S5E,F).

**Figure 5:**
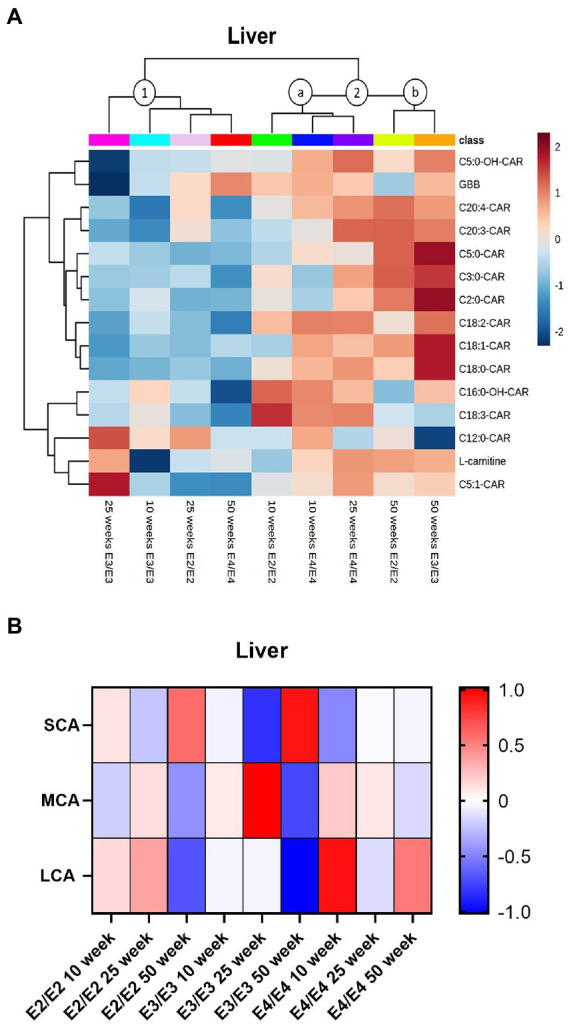
Liver acylcarnitines are affected by *APOE* and sex. **(A)** Hierarchical clustering heatmap showing acylcarnitine species with *APOE* genotypes at different age timepoints in the liver. **(B)** Z-score heatmap showing acylcarnitine chain length ratios to total acylcarnitines with *APOE* genotypes at different age timepoints in the liver. Statistics: For the clustering heatmap data were normalized and scaled before being analyzed using Ward’s clustering, with the top 15 acylcarnitines selected by ANOVA. Multiple comparisons with B-H correction were performed on the data. Numbers per group; 10-week APOE2-TR *n* = 6, 25-week APOE2-TR *n* = 5, 50-week APOE2-TR *n* = 6, 10-week APOE3-TR *n* = 6, 25-week APOE3-TR *n* = 5, 50-week APOE3-TR *n* = 6, 10-week APOE4-TR *n* = 6, 25-week APOE4-TR *n* = 6, 50-week APOE4-TR *n* = 4. Cx:y-CAR, acylcarnitines; Cx:y-OH-CAR, hydroxy acylcarnitines; GBB, γ-butyrobetaine; LCA, long chain acylcarnitines; MCA, medium chain acylcarnitines; SCA, short chain acylcarnitines.

In the liver, two major clusters and two sub-clusters could be delineated based on acylcarnitine profiles (clusters 1, 2a, and 2b, Figure 5A). The first cluster contained 50- and 25-weeks old E2 mice, as well as 10- and 25-week E3 mice, which had overall lower levels of acylcarnitines (cluster 1, Figure 5A). The second cluster composed of two subclusters: in the first subcluster 10- and 25-week E4 mice and 10-week E2 mice, with trends for increases in MCAs in all groups (cluster 2a, Figure 5A). The second subcluster was constituted of 50-week-old E2 and E3 mice (cluster 2b, Figure 5A). There was no significant interaction of age and sex on liver total acylcarnitines. While total acylcarnitines did not significantly increase with age in the liver (Supplementary Figures S6A,C,D), a significant increase in SCAs was detected with age in all genotypes (*p* = 0.039, Figure 5B). There were independent effects of *APOE* and sex as well as interactions between them for LCAs (*p* < 0.05, Figure 5B). L-carnitine, GBB, TMAO and most acylcarnitines were significantly increased in females compared to males (Supplementary Figure S4B). As with brain and plasma, TMAO was higher in E3 males compared to males from other genotypes (Supplementary Figures S6E,F).

**Figure 6:**
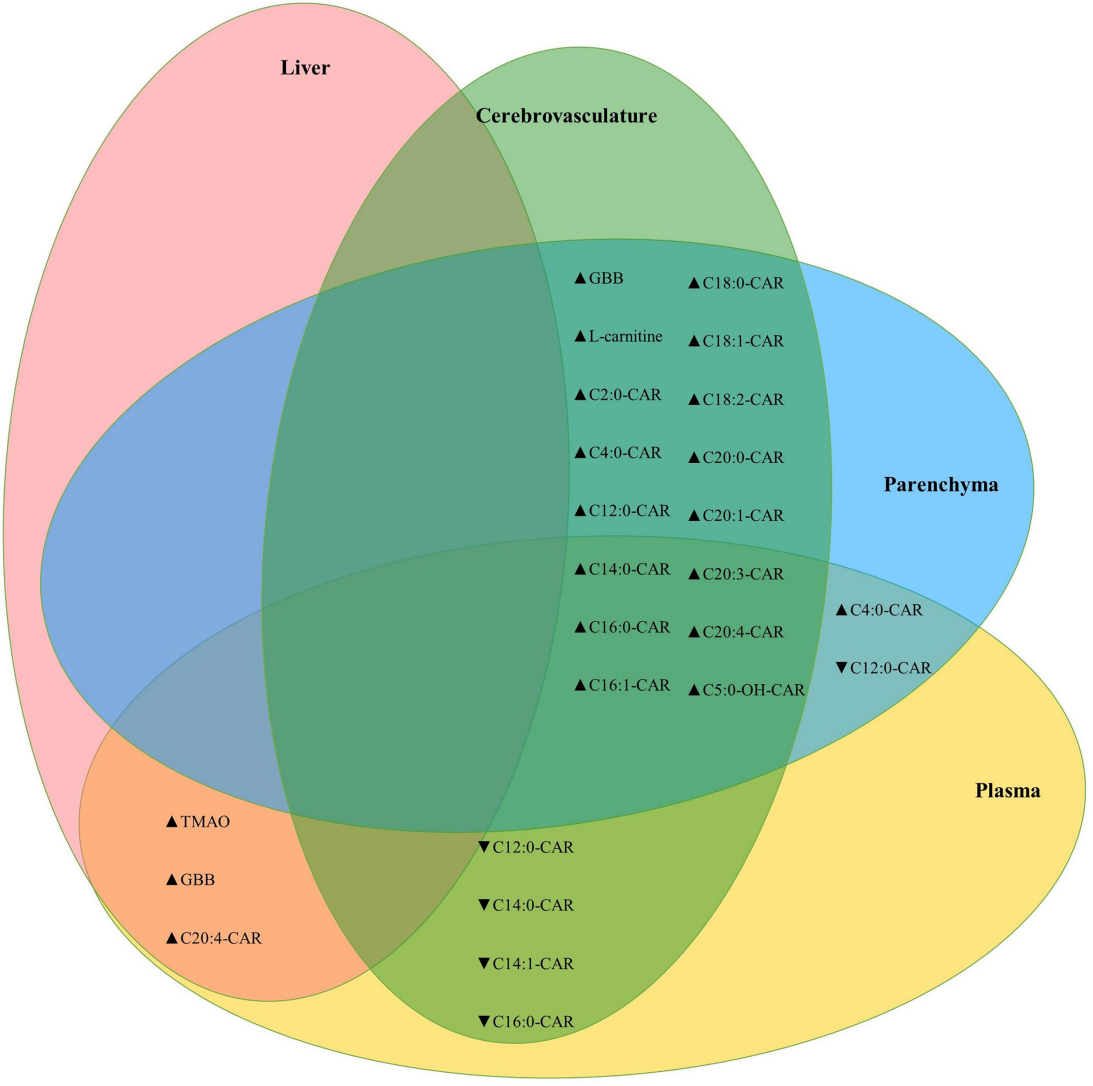
Cerebrovascular and brain parenchymal acylcarnitines are highly positively correlated. Venn diagram showing significant correlations between parenchymal, cerebrovascular, plasma, and liver L-carnitine, GBB, and acylcarnitines. The TMAO metabolite was also correlated between plasma and brain homogenate (Kendall’s tau-b = 0.386, *p* = 7.6E-5) and between brain homogenate and liver (Kendall’s tau-b = 0.383, *p* = 8.7E-5). Statistics: Kendall’s tau-b with two-tailed significance threshold *p* < 0.05, ▲: positive correlation, ▼: negative correlation. Cx:y-CAR, acylcarnitines; Cx:y-OH-CAR, hydroxy acylcarnitines; GBB, γ-butyrobetaine; TMAO, trimethylamine-n-oxide.

Between tissue correlation analyses were performed for L-carnitine system metabolites to assess how these relate to each other. L-carnitine, GBB, and acylcarnitines were highly correlated between the brain parenchyma and cerebrovascular fractions, while the MCA C12:0-CAR and three additional LCA species were negatively correlated between the cerebrovasculature and plasma. Levels of TMAO were also positively correlated across all tissues measured (brain, plasma, and liver; Figure 6).

### *APOE* does not affect the cerebrovascular uptake of FAO-related metabolites

**Figure 7:**
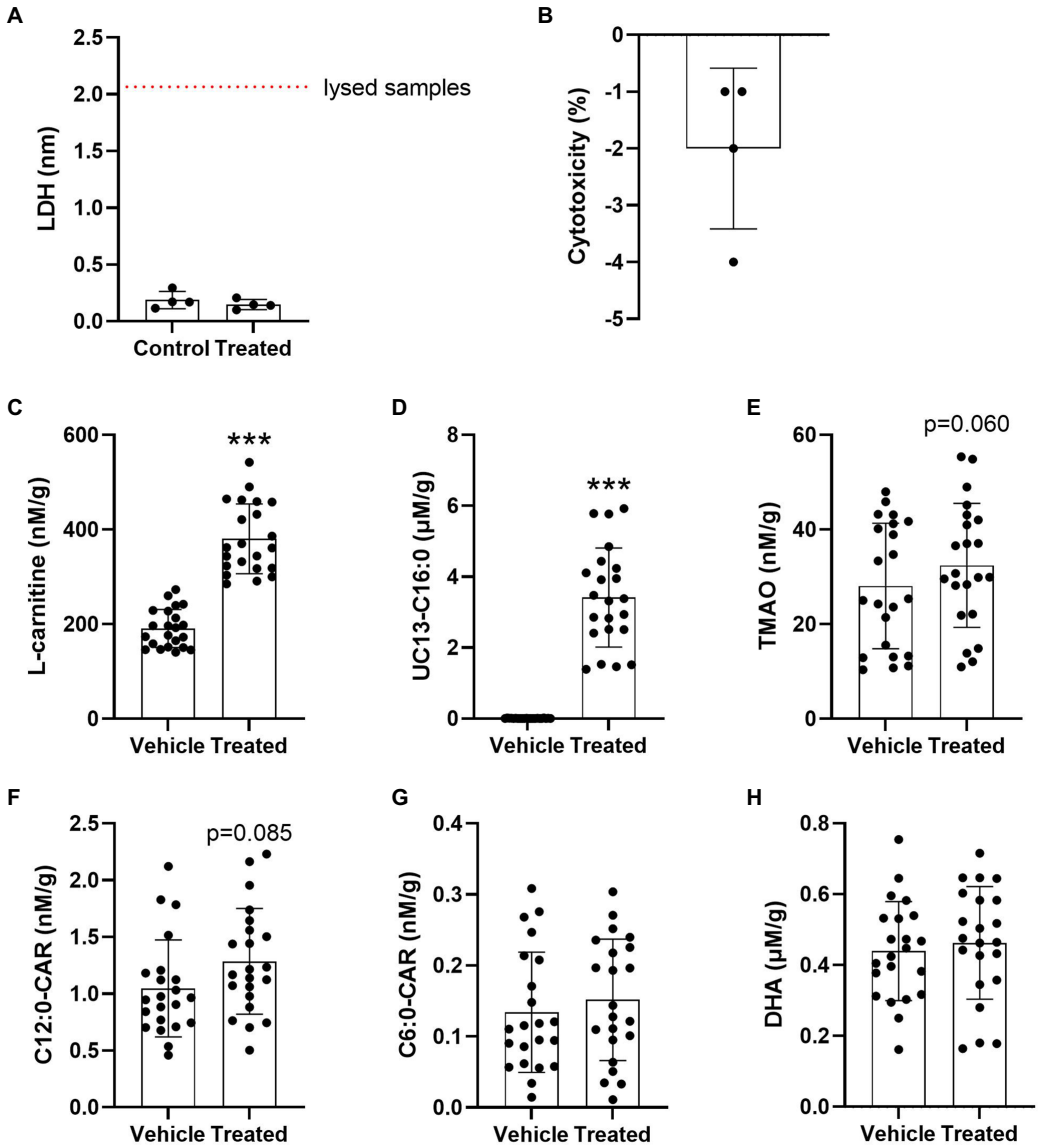
Significant cerebrovascular uptake of treatment compounds. **(A)** Scatterplots with bar showing mean LDH absorbance values in nm in vehicle treated and compound treated cerebrovascular fractions. **(B)** Scatterplots with bar showing % treatment cytotoxicity ± SD. **(C)** Scatterplots with bar showing mean concentration of L-carnitine in nM per gram of protein ± SD in vehicle treated and compound treated cerebrovasculature. **(D)** Scatterplots with bar showing mean concentration of UC13-C16:0 in μM per gram of protein ± SD in vehicle treated and compound treated cerebrovasculature. **(E)** Scatterplots with bar showing mean concentration of TMAO in nM per gram of protein ± SD in vehicle treated and compound treated cerebrovasculature. **(F)** Scatterplots with bar showing mean concentration of C12:0-CAR in nM per gram of protein ± SD in vehicle treated and compound treated cerebrovasculature. **(G)** Scatterplots with bar showing mean concentration of C6:0-CAR in nM per gram of protein ± SD in vehicle treated and compound treated cerebrovasculature. **(H)** Scatterplots with bar showing mean concentration of DHA in μM per gram of protein ± SD in vehicle treated and compound treated cerebrovasculature. Statistics: related-samples analysis, ***p < 0.001. Numbers per group: matched control-treated cerebrovascular sample pairs *n* = 4 per group for LDH test. Matched control-treated cerebrovascular sample pair *n* = 22 per group for the uptake study. Cx:y-CAR, acylcarnitines; DHA, docosahexaenoic acid; LDH, lactate dehydrogenase; TMAO, trimethylamine-n-oxide; UC13-C16:0, uniformly C13-labeled palmitic acid.

Due to the evidence of disturbed brain transport in E4s we next investigated the cerebrovascular uptake of L-carnitine, TMAO, FAs (precursors to acylcarnitines), and the MCAs C6:0-CAR and C12:0-CAR in 26-week-old in APOE-TR mice cerebral vessels. No significant treatment-related cytotoxicity was detected (Figures 7A,B). There was a significant effect of treatment for L-carnitine and the UC13-C16:0 (Figures 7C,D). No further independent effects of treatment or *APOE* were seen for other compounds (Figures 7E–H) and no interactive effect of treatment and *APOE* (Supplementary Figures S7A–F). Additional treatment and *APOE* effects on cerebrovascular acylcarnitines can be found in Supplementary Figures S8A–E, S9A–C.

### *APOE* and sex are associated with changes in TMA-containing metabolites following L-carnitine challenge in mid-life mice

Given the association between L-carnitine gut-related metabolites and *APOE* with cardiovascular disease, we investigated the effect of different *APOE* genotypes on the metabolism of L-carnitine, acylcarnitines, and other metabolites capable of producing TMA and TMAO (TMA-containing metabolites) to investigate whether changes in TMA/TMAO could be associated with either L-carnitine or other metabolites capable of producing these metabolites after an L-carnitine challenge. No behavioral differences or signs of adverse effects were observed between vehicle and L-carnitine challenged groups and liver AST levels also did not significantly differ between groups indicating that L-carnitine challenge did not lead to significant hepatotoxicity (Supplementary Figure S10A). The E4 group had significantly lower body weight than other genotypes regardless of vehicle or L-carnitine challenge, although the E4 group was 6 weeks younger on average, the age covariate had no significant effect on weight (Supplementary Figure S10B).

**Figure 8:**
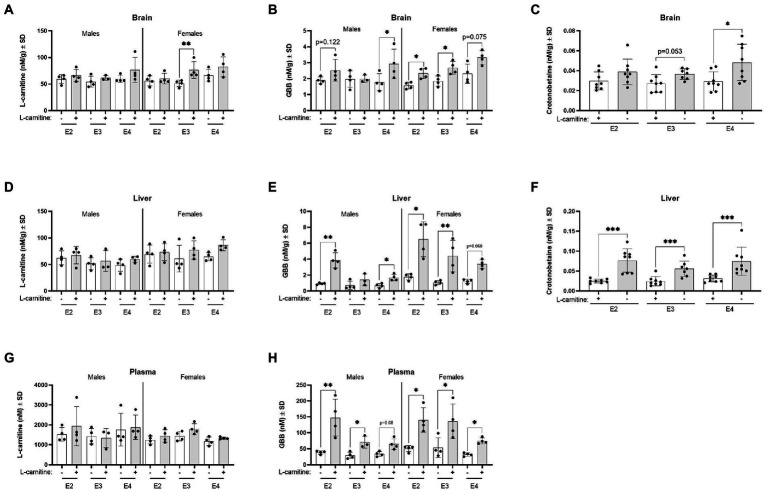
L-carnitine metabolites are affected by sex and *APOE* after an oral L-carnitine challenge. **(A)** Scatterplots with bar showing mean concentration of L-carnitine in nM per gram of protein ± SD in the brain of vehicle and L-carnitine challenged mice with sex and *APOE* genotype. **(B)** Scatterplots with bar showing mean concentration of GBB in nM per gram of protein ± SD in the brain of vehicle and L-carnitine challenged mice with sex and *APOE* genotype. **(C)** Scatterplots with bar showing mean concentration of crotonobetaine in nM per gram of protein ± SD in the brain of vehicle and L-carnitine challenged mice in different *APOE* genotypes. **(D)** Scatterplots with bar showing mean concentration of L-carnitine in nM per gram of protein ± SD in the liver of vehicle and L-carnitine challenged mice with sex and *APOE* genotype. **(E)** Scatterplots with bar showing mean concentration of GBB in nM per gram of protein ± SD in the liver of vehicle and L-carnitine challenged mice with sex and *APOE* genotype. **(F)** Scatterplots with bar showing mean concentration of crotonobetaine in nM per gram of protein ± SD in the liver of vehicle and L-carnitine challenged in different *APOE* genotypes. **(G)** Scatterplots with bar showing mean concentration of L-carnitine in nM ± SD in plasma in vehicle and L-carnitine challenged mice with sex and *APOE* genotype. **(H)** Scatterplots with bar showing mean concentration of GBB in nM ± SD in plasma in vehicle and L-carnitine challenged mice with sex and *APOE* genotype. Statistics: multiple comparisons with B-H correction, *p < 0.05, **p < 0.01, ***p < 0.001. Numbers per group: male E2 (vehicle) *n* = 4, male E2 (L-carnitine) *n* = 4, male E3 (vehicle) *n* = 4, male E3 (L-carnitine) *n* = 3, male E4 (vehicle) *n* = 4, male E4 (L-carnitine) *n* = 4, female E2 (vehicle) *n* = 4, female E2 (L-carnitine) *n* = 4, female E3 (vehicle) *n* = 4, female E3 (L-carnitine) *n* = 4, female E4 (vehicle) *n* = 4, female E4 (L-carnitine) *n* = 4, E2/E2 (vehicle) *n* = 8, E2/E2 (L-carnitine) *n* = 8, E3/E3 (vehicle) *n* = 8, E3/E3 (L-carnitine) *n* = 7, E4/E4 (vehicle) *n* = 8, E4/E4 (L-carnitine) *n* = 8. GBB, γ-butyrobetaine. Crotonobetaine not detected in plasma.

Irrespective of *APOE* genotype, L-carnitine challenge increased brain L-carnitine and crotonobetaine levels (Supplementary Figures S11A,B). In the brain, there was a significant influence of *APOE* genotypes on L-carnitine and GBB which was further mediated by sex. Brain L-carnitine levels significantly increased among female E3 mice after the L-carnitine challenge compared to female E3 vehicle challenged mice (Figure 8A). The highest increase was seen for brain GBB among E4 males and E2 and E3 females after L-carnitine challenge (Figure 8B). In the brain, crotonobetaine levels were significantly increased in E4 mice following L-carnitine challenge with no significant influence of sex (Figure 8C). In the liver, L-carnitine, GBB, and crotonobetaine levels were also increased following challenge (Supplementary Figure S11C; Figures 8E,F) and there was no influence of *APOE* genotypes or sex (Figures 8D,F). For liver GBB, the most significant increase after challenge was seen in male E2 mice and female E3 mice compared to their respective sex (Figure 8E). In plasma, while L-carnitine levels did not significantly increase following challenge, GBB levels were increased in both male and female mice across all genotypes, with most significant increases detected in male E2 mice (Figures 8G,H). There were no significant changes in the levels of TML, TMA, betaine, TMAO, and choline in different *APOE* genotype groups after L-carnitine challenge (all *p* > 0.05, data not shown).

**Figure 9:**
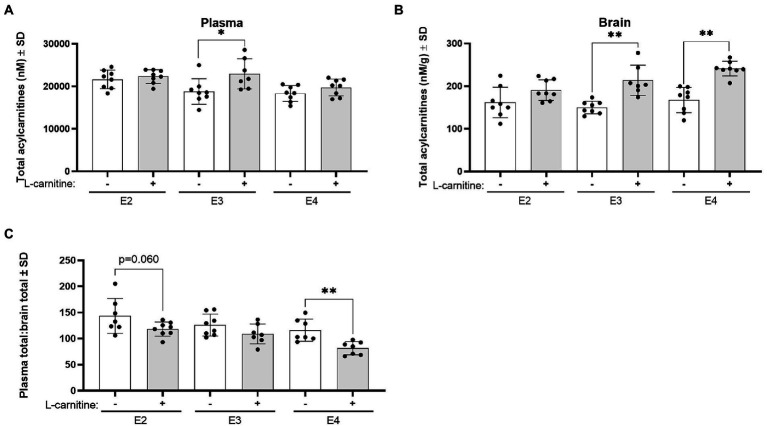
*APOE* genotype and L-carnitine challenge affect plasma total acylcarnitines to brain ratios. **(A)** Scatterplots with bar showing mean plasma concentration of total acylcarnitines in nM ± SD in vehicle and L-carnitine challenged mice in different *APOE* genotypes. **(B)** Scatterplots with bar showing mean brain concentration of total acylcarnitines in nM per gram of protein ± SD in vehicle and L-carnitine challenged mice in different *APOE* genotypes. **(C)** Scatterplots with bar showing mean plasma total acylcarnitines to brain total acylcarnitines ratio ± SD in vehicle and L-carnitine challenged mice in different *APOE* genotypes. Statistics: multiple comparisons with B-H correction, *p < 0.05, **p < 0.01, ***p < 0.001. Numbers per group: E2/E2 (vehicle) *n* = 8, E2/E2 (L-carnitine) *n* = 8, E3/E3 (vehicle) *n* = 8, E3/E3 (L-carnitine) *n* = 7, E4/E4 (vehicle) *n* = 8, E4/E4 (L-carnitine) *n* = 8.

Changes in total acylcarnitine levels were also examined in the plasma and brain following challenge. In plasma, L-carnitine challenge was associated with significantly increased total acylcarnitine levels in E3 mice but not in other genotypes (Figure 9A). In the brain, L-carnitine challenge was accompanied by an increase in total acylcarnitines in both E3 and E4 mice but not in E2 mice (Figure 9B). An examination of the ratios of plasma to brain total acylcarnitine levels showed a significant decrease in this ratio in E4 mice after challenge with L-carnitine but not in E3 mice, while there was a trend in E2 mice (Figure 9C).

In addition, to the interactive effect of sex and *APOE* described above there were strong sex effects observed on L-carnitine gut metabolites, and other TMA-containing metabolites, and acylcarnitines in the plasma,brain, and liver (Supplementary Table S7).

Correlations between brain, liver, and plasma L-carnitine, its metabolites, and acylcarnitines are presented in Supplementary Figure S12.

## Discussion

The L-carnitine system is essential to mitochondrial FAO and is a major source of acetyl-CoA necessary for ketogenesis (Jones et al., 2010). It also plays an important role in maintaining the intracellular free CoA pool, when excess intra-mitochondrial acyl- groups are present, through the reverse transport of fatty acyl groups as acylcarnitines by the L-carnitine system (Figure 1; Jones et al., 2010). These acyl- groups can come from mitochondrial FAO, amino acids catabolism, and the peroxisomal FAO of very long chain fatty acids (Wanders et al., 2016). As such, acylcarnitines levels in biological samples reflect the metabolism of acyl- groups for energy from various sources and can inform on current metabolic state which is why they serve as biomarkers of metabolic disorders (Rinaldo et al., 2008; Schooneman et al., 2013). Current literature suggests that the L-carnitine system is perturbed in AD, with multiple studies showing that proteins and metabolites related to the L-carnitine system are altered in the brain, CSF, and plasma of AD patients (Makar et al., 1995; González-Domínguez et al., 2014; Mapstone et al., 2014; Ciavardelli et al., 2016; Cristofano et al., 2016; Toledo et al., 2017; Varma et al., 2018; Lin et al., 2019; Van Der Velpen et al., 2019; Arnold et al., 2020; Huo et al., 2020; Lim et al., 2020; Baloni et al., 2021; Horgusluoglu et al., 2021; Nho et al., 2022). However, relatively few studies have been able to examine both plasma and brain acylcarnitines in relation to *APOE* and age. A comprehensive metabolomics and transcriptomics study investigating bioenergetics, including brain and plasma acylcarnitines, in 16 months old APOE3-TR and APOE4-TR mice found increases in FAO in E4s especially in females (Shang et al., 2020). Although prior studies have shown that an impairment of FAO in the cerebrovasculature can result in defects in the cerebrovascular transport system (Steinberg et al., 1977) very little is known about the L-carnitine system in the cerebrovasculature in relation to *APOE* and age. In AD, cerebrovascular dysfunction occurs earlier among E4 carriers (Veitch et al., 2019), which could lead to impairments in nutrient sensing and metabolite exchange between the brain and periphery, thereby negatively impacting brain bioenergetics by rendering brain areas of high energetic needs (e.g., the hippocampus) vulnerable to pathologies such as amyloid (Veitch et al., 2019; Arvanitakis et al., 2021). Understanding the L-carnitine system in relation to *APOE* genotype with a focus on the cerebrovasculature is important due to its crucial role in delivering nutrients from the periphery to the brain. The work described below is aimed at addressing these outstanding questions to better understand brain bioenergetic changes that precede AD pathology.

Our studies of the effects of age and *APOE* in the cerebrovasculature showed that with age, total acylcarnitine levels increased in the cerebrovasculature of E2 and E3 mice but not in E4 mice, this was also observed in the brain parenchyma. As E2 and E3 mice are known to have increased longevity relative to E4 mice (Shinohara et al., 2020), this overall increase in cerebrovascular acylcarnitines could represent compensatory changes to supplement energetics with age, or act as a FA detoxification mechanism, a response which appears blunted in E4s. Since LCFAs preferentially undergo FAO in mitochondria, alterations in LCA levels may reflect changes in mitochondrial FAO in E4s with age compared to E2 and E3 mice. Differences in FAO in APOE4-TR mice compared to other genotypes has been suggested in the periphery and in the brain in E4 mice (Arbones-Mainar et al., 2016; Shang et al., 2020; Qi et al., 2021), however, to our knowledge this had not previously been shown in the cerebrovasculature.

Although ApoE4 has been shown to disrupt Blood Brain Barrier (BBB) integrity and reduce its glucose uptake (Brandon et al., 2018), relatively few studies have investigated the effect of ApoE on cerebrovascular FAO bioenergetics. A recent study in E4 carriers with and without AD found evidence of disturbed mitochondrial function regardless of disease phenotype in the cerebrovasculature of E4 carriers compared to other alleles (Ojo et al., 2021). The current study did not find an *APOE* genotype effect on the cerebrovascular uptake of L-carnitine, its metabolites, or FAs at 26 weeks of age. Although in cognitively healthy individuals differences in BBB uptake have been observed with *APOE* genotypes as early as in individuals in their 30s (Yassine et al., 2017) in mice older ages may be required for such disturbances to occur (Alata et al., 2015). These studies are the first to investigate age-dependent changes in the L-carnitine system as a surrogate for FAO within the cerebrovasculature from mice with different human *APOE* genotypes. Cerebral micro-vessels are able to use both glucose and FAs as energy sources, a process seemingly driven by nutrient availability (Hingorani and Brecher, 1987). However, inhibition of FAO in the cerebrovasculature even in the presence of glucose leads to defective cation transport (Steinberg et al., 1977). The cerebrovasculature is a very bioenergetically active tissue with capillary endothelial cells having been shown to possess five times more mitochondria than muscle cells (Oldendorf and Brown, 1975) and transport 10 times their weight in glucose each minute across the BBB, along with accomplishing other energetically costly cellular processes (Cunnane et al., 2011). Therefore, E4-related bioenergetic dysfunction, such as impairments in FAO of the cerebrovasculature, could have detrimental impacts on its transport functions. Studies in older mice investigating individual cells as well as larger vessels and micro-vessels will be useful in determining which cells of the cerebrovasculature are responsible for these changes and whether there are *APOE*-driven differences in FAO in both larger and smaller vessels that can affect transport functions.

Age-related brain cerebrovascular and brain parenchymal changes observed were also accompanied by changes in the periphery, with E2 mice retaining profiles similar to that of younger mice of other genotypes even as they age. Unlike E3 and E4 mice, in E2 mice plasma acylcarnitines, particularly LCAs, remained elevated with age and overall higher than in other genotypes regardless of age. Interestingly, a human study has shown increases in plasma acylcarnitines with healthy aging (Jarrell et al., 2020). Conversely, in E4 mice SCAs were higher compared to other genotypes. These findings are similar to data recently published by us in clinical cohorts, with E4 carriers exhibiting relatively higher SCAs and lower LCAs with AD even at the pre-clinical stages compared to controls and other *APOE* genotypes (Huguenard et al., 2022). This is also in line with a study in older 16 months old APOE-TR mice which found the same pattern of decreased LCAs and increased SCAs in E4s compared to E3s, particularly in males (Shang et al., 2020). Decreases in LCAs and MCAs in E4s may reflect poor mitochondrial FAO capacity or alternatively increased cellular reliance on FAs to support bioenergetics in the cerebrovasculature. This could also have detrimental effects on FA buffering capacity in E4s. Lastly, while *APOE* effects dominated brain profiles, sex effects were more prominent in the periphery, which was also in accordance with a previous study in older (16 months old) APOE-TR mice (Shang et al., 2020), suggesting that regardless of age *APOE* has a major effect on brain bioenergetics. Collectively, understanding the relationship between acylcarnitine levels in different tissues and how these reflect mitochondrial (as well as peroxisomal) dynamics warrants further investigation.

In addition to its biosynthesis, L-carnitine can also be obtained from the diet, where in the gut L-carnitine can either be directly absorbed or converted to GBB or crotonobetaine by bacteria residing in more distal gut segments. L-carnitine, GBB, and crotonobetaine can also be further converted to TMA by bacteria, with TMA itself being subsequently absorbed and processed to TMAO by liver enzymes (Figure 1; Koeth et al., 2013; Meadows and Wargo, 2015; Koeth et al., 2019). L-carnitine metabolism to GBB, crotonobetaine, and TMA (and ultimately TMAO) could affect L-carnitine availability for bioenergetics, particularly if biosynthesis is also impaired and/or there is an increased reliance in FAO for energy production (i.e., an increased demand for L-carnitine). Conversely changes in L-carnitine gut metabolism could also reflect alterations in its endogenous use as part of the L-carnitine system. Consistently with prior studies of L-carnitine administration in mice (Koeth et al., 2014), in our study, we observed that peripheral GBB, and to a lesser extent crotonobetaine, increased in mice after L-carnitine oral challenge but not TMAO. We, for the first time, show that an L-carnitine oral challenge increases brain GBB levels in E2 and E4 mice (but not in E3 males) and brain crotonobetaine only in E4 mice. Since GBB and crotonobetaine after L-carnitine challenge are likely primarily generated in the gut, these brain increases are likely to be secondary to increased transport of these metabolites. Crotonobetaine, to our knowledge, has not been measured in the brain, while peripheral crotonobetaine levels have been associated with an increased risk of cardiovascular events as well as increased fasting insulin levels (Koeth et al., 2014; Lemaitre et al., 2021), its role in the brain, particularly in relation to *APOE* genotypes has not been yet explored. Given that E2 and E4 alleles are associated with vascular diseases (Belloy et al., 2019) and both GBB and crotonobetaine have been associated with vascular disease (Koeth et al., 2014, 2019; Lemaitre et al., 2021), additional studies are required to better understand the potential role of *APOE* genotype on modulating cerebrovascular risk *via* these L-carnitine gut-related metabolites.

Peripheral TMAO levels were higher in E3 mice compared to other genotypes; these genotype differences also seemed stronger in males. This was an unanticipated finding since TMAO is associated with cardiovascular and metabolic disease and AD and prior human studies showed modulation of plasma TMAO by the E4 allele (Fennema et al., 2016; Vogt et al., 2018; Buawangpong et al., 2021; Huguenard et al., 2022). The E4 allele itself is also associated with cardiovascular disease and metabolic syndrome (Belloy et al., 2019); therefore it is unclear why TMAO was found increased in E3 mice both peripherally and in the brain compared to E4 mice. While most studies suggest that TMAO is an atherogenic agent, some studies have also demonstrated protective vascular effects of TMAO in addition to its known osmolyte function (Jethva and Udgaonkar, 2018; Gawrys-Kopczynska et al., 2020; Hoyles et al., 2021). Clearly more research on TMAO in different disease context as well as longitudinal studies are needed to understand the potential impact of this metabolite on biological systems in health and disease. Since the L-carnitine used in these studies was not labeled, we could not determine how much of the administered L-carnitine and its metabolites distributed to different tissues in the body. Importantly, in mice GBB is the major metabolite produced from L-carnitine gut metabolism, while in humans TMAO appears to be the major metabolite following L-carnitine challenge (Koeth et al., 2014). Nevertheless, since both metabolites are associated with vascular dysfunction and both are produced in mice and humans following L-carnitine challenge, studying these compounds will allow for an examination of their role in the cerebrovascular dysfunction associated with E4. Further studies are also needed in humans to determine whether *APOE* also influences L-carnitine metabolism following challenge and whether this impacts GBB and/or TMAO levels.

L-carnitine challenge also increased total acylcarnitine levels in both plasma and brain in E3 mice, while brain (but not plasma) total acylcarnitines were also significantly increased by the challenge in E4 mice. These findings point to a potential increased transport to and/or production of acylcarnitines within the brain of E4 mice following challenge. As different *APOE* genotypes are shown to have different energy substrate preferences it is not surprising that L-carnitine administration affected levels of these FAO metabolites differently by genotype (Yassine and Finch, 2020).

Several sex effects were also observed throughout these studies, where despite higher levels of total plasma acylcarnitines in females compared to males, in aging females, there was a significant decrease in total acylcarnitines which was not seen in aging males, indicating that aging may impact female peripheral lipid bioenergetics more than males. A clinical study has shown that female E4 carriers who experienced late life weight loss were at an increased risk of developing dementia but not males (Kristoffer et al., 2015). There were also expected sex differences in TMA-containing compounds in mice with higher TMAO but lower TMA in females compared to males in plasma, liver, and brain. In mice plasma TMAO has been shown to be higher in female and TMA lower compared to males as Flavin-Containing Monooxygenase 3 (the main TMAO producing enzyme) expression is drastically downregulated by testosterone (and mildly upregulated by estrogen; Bennett et al., 2013). Higher, levels of betaine were also observed in the plasma, liver, and brain in females compared to males. This is in contrast with what is seen in clinical populations where higher plasma betaine has been found in males compared to females (Lever et al., 1994, 2007; Barnes et al., 2010). In the liver L-carnitine, and GBB were higher and choline lower in females compared to males. This again differs from findings in humans where in plasma L-carnitine and GBB levels have been observed to be lower in females (Reuter et al., 2008; Gao et al., 2018). However the lower choline observed in female mice in our studies compared to male mice is consistent with sex differences found in humans (Andraos et al., 2020). These findings indicate differences in sex effects on L-carnitine metabolism and acylcarnitines between mice and humans. Lastly, multiple plasma and brain acylcarnitines were affected by sex across studies, however, the directionality of these changes did not stay consistent across studies, although *APOE* effect such as that on TMAO did.

In conclusion, these studies showed an effect of *APOE* on L-carnitine, its metabolites and acylcarnitines with age in the brain parenchyma and in the cerebrovasculature up to mid-life, which suggest differences in age-related bioenergetic shift between alleles. In particular, the E4 allele mediated decline in MCA and LCAs within the cerebrovasculature at 50 weeks may indicate that cerebrovascular mitochondrial FAO flux is impaired earlier in E4 relative to other alleles, especially in female E4s who fail to increase acylcarnitines with age, and at older ages in the same animal model have been shown to fail to increase AA as an alternative energy source compared to male E4s (Shang et al., 2020). In aging mice this could mean a failure to adapt to aging-related bioenergetics deficits with less flexibility in fuel use in E4s which is further aggravated by female sex. L-carnitine challenge studies also showed that *APOE* had an impact on the production of L-carnitine metabolites associated with vascular dysfunction. Additionally, following challenge E4 mice preserved a higher proportion of acylcarnitine in the brain relative to the periphery compared to E2 and E3 mice. Collectively, these studies provide evidence that the L-carnitine system is differentially impacted by *APOE* genotypes with age and following L-carnitine challenge which could later contribute to AD pathogenesis through bioenergetics deficits, FA toxicity, and cerebrovascular dysfunction in E4s, especially in females. As such, future studies investigating the role of the L-carnitine system in mediating mitochondrial bioenergetics and FA buffering in relation to AD and *APOE* genotypes will be needed to develop approaches for targeting this system and potentially mitigating the risk of AD in different *APOE* genotypes particularly in females.

## Data availability statement

The original contributions presented in the study are included in the article/Supplementary material, further inquiries can be directed to the corresponding author.

## Ethics statement

The animal study was reviewed and approved by the Roskamp Institute IACUC.

## Author contributions

CH wrote the manuscript. LA, SH, and JE reviewed and edited the manuscript. AC, JE, and CH developed/performed the LC–MS/MS method and assisted with the experiments. TD and AN assisted with the sample processing and experiments. CH, LA, MM, FC, and JE assisted with data interpretation. LA designed the study. FC advised on the study design. All authors contributed to the article and approved the submitted version.

## Funding

This work was supported by an NIH (R03AG070540-0) and a VA Merit award (BX004352-01) to LA. FC is a VA Research Career Scientist.

## Conflict of interest

SH reports being supported by a grant from the NIH and Office of Dietary Supplements (R01HL103866). SH, reports being named as co-inventor on pending and issued patents held by the Cleveland Clinic relating to cardiovascular diagnostics and/or therapeutics and being eligible to receive royalty payments for inventions or discoveries related to cardiovascular diagnostics or therapeutics from Cleveland HeartLab, a wholly owned subsidiary of Quest Diagnostics, P&G, and Zehna Therapeutics. SH also reports being a paid consultant for Zehna Therapeutics, having received research funds from P&G, Roche Diagnostics, and Zehna Therapeutics.

The remaining authors declare that the research was conducted in the absence of any commercial or financial relationships that could be construed as a potential conflict of interest.

## Publisher’s note

All claims expressed in this article are solely those of the authors and do not necessarily represent those of their affiliated organizations, or those of the publisher, the editors and the reviewers. Any product that may be evaluated in this article, or claim that may be made by its manufacturer, is not guaranteed or endorsed by the publisher.

## Author disclaimer

The content of this article is solely the responsibility of the authors and does not necessarily represent the official views of the National Institutes of Health, the Department of Veterans Affairs or the United States Government.

## Figures and Tables

**Table 1:** 

AA	Acetic Acid
ACN	Acetonitrile
AD	Alzheimer’s Disease
AmFm	Ammonium Formate
*APOE*	Apolipoprotein (gene)
ApoE	Apolipoprotein (protein)
APOE-TR	*APOE*-targeted replacement
AST	Aspartate Aminotransferase
BBB	Blood Brain Barrier
B-H	Benjamini-Hochberg
Cx:y	free fatty acid
Cx:y-CAR	acylcarnitine
Cx:y-DC-CAR	dicarboxy acylcarnitine
Cx:y-OH-CAR	hydroxy acylcarnitine
CV	Coefficient of Variance
FAO	Fatty Acid Oxidation
GBB	γ-butyrobetaine
HILIC	Hydrophilic Interaction Liquid Chromatography
IPA	Isopropanol
IS	Internal Standard
LCA	Long Chain Acylcarnitine
LCFA	Long Chain Fatty Acid
LDH	Lactate Dehydrogenase
LSD	Least Significant Difference
MCA	Medium Chain Acylcarnitine
MeOH	Methanol
MLM	Mixed Linear Model
M-PER	Mammalian Protein Extraction Reagent
OCA	Odd Chain Acylcarnitine
RCF	Relative Centrifugal Force
SCA	Short Chain Acylcarnitine
TMA	Trimethylamine
TMAO	Trimethylamine-n-oxide
TML	Trimethyl-l-lysine
UC13-C16:0	Uniformly C13-labeled palmitic acid
VLCA	Very Long Chain Acylcarnitine

## Footnotes

^1^ https://www.metaboanalyst.ca/

^2^ https://biorender.com/

## References

[ref1] Alata W. Ye Y. St-Amour I. Vandal M. Calon F. (2015). Human apolipoprotein E ε4 expression impairs cerebral vascularization and blood-brain barrier function in mice. J. Cereb. Blood Flow Metab. 35, 86–94. doi: 10.1038/jcbfm.2014.172, PMID: 25335802 PMC4296574

[ref2] Andraos S. Lange K. Clifford S. A. Jones B. Thorstensen E. B. Kerr J. A.. (2020). Plasma trimethylamine N-oxide and its precursors: population epidemiology, parent–child concordance, and associations with reported dietary intake in 11- to 12-year-old children and their parents. Curr Dev Nutr. 4, 1–11. doi: 10.1093/cdn/nzaa103/5855489 32666035 PMC7335361

[ref3] Arbones-Mainar J. M. Johnson L. A. Torres-Perez E. Garcia A. E. Perez-Diaz S. Raber J.. (2016). Metabolic shifts toward fatty-acid usage and increased thermogenesis are associated with impaired adipogenesis in mice expressing human APOE4. Int. J. Obes. 40, 1574–1581. doi: 10.1038/ijo.2016.93, PMID: 27163745 PMC5063049

[ref4] Arnold M. Nho K. Kueider-Paisley A. Massaro T. Huynh K. Brauner B.. (2020). Sex and APOE ε4 genotype modify the Alzheimer’s disease serum metabolome. Nat. Commun. 11:1148. doi: 10.1038/s41467-020-14959-w, PMID: 32123170 PMC7052223

[ref5] Arvanitakis Z. Capuano A. W. Wang H. Y. Schneider J. A. Kapasi A. Bennett D. A.. (2021). Brain insulin signaling and cerebrovascular disease in human postmortem brain. Acta Neuropathol. Commun. 9, 71–79. doi: 10.1186/s40478-021-01176-9, PMID: 33858515 PMC8048276

[ref6] Baloni P. Nho K. Arnold M. Louie G. Kueider-Paisley A. Saykin A. J.. (2021). Investigating the importance of acylcarnitines in Alzheimer’s disease. Alzheimers Dement. 17:e056647. doi: 10.1002/alz.056647

[ref7] Barnes M. S. McAfee A. Bonham M. P. McSorley E. M. Wallace J. M. W. Myers G. J.. (2010). Age and sex differences in plasma homocysteine, choline and betaine status in Seychellois children and young adults. Proc. Nutr. Soc. 69:E381. doi: 10.1017/S0029665110002429

[ref8] Belloy M. E. Napolioni V. Greicius M. D. (2019). A quarter century of APOE and Alzheimer’s disease: Progress to date and the path forward. Neuron 101, 820–838. doi: 10.1016/j.neuron.2019.01.056, PMID: 30844401 PMC6407643

[ref9] Bennett B. J. Vallim T. Q. D. A. Wang Z. Shih D. M. Meng Y. Gregory J.. (2013). Trimethylamine-N-oxide, a metabolite associated with atherosclerosis, exhibits complex genetic and dietary regulation. Cell Metab. 17, 49–60. doi: 10.1016/j.cmet.2012.12.011, PMID: 23312283 PMC3771112

[ref10] Brandon J. A. Farmer B. C. Williams H. C. Johnson L. A. (2018). APOE and alzheimer’s disease: neuroimaging of metabolic and cerebrovascular dysfunction. Front. Aging Neurosci. 10:180. doi: 10.3389/fnagi.2018.00180, PMID: 29962946 PMC6010552

[ref11] Buawangpong N. Pinyopornpanish K. Siri-Angkul N. Chattipakorn N. Chattipakorn S. C. (2021). The role of trimethylamine-N-oxide in the development of Alzheimer’s disease. J. Cell. Physiol. 237, 1661–1685. doi: 10.1002/jcp.30646 34812510

[ref12] Ciavardelli D. Piras F. Consalvo A. Rossi C. Zucchelli M. Di Ilio C.. (2016). Medium-chain plasma acylcarnitines, ketone levels, cognition, and gray matter volumes in healthy elderly, mildly cognitively impaired, or Alzheimer’s disease subjects. Neurobiol. Aging. 43, 1–12. doi: 10.1016/j.neurobiolaging.2016.03.005 27255810

[ref13] Cristofano A. Sapere N. La Marca G. Angiolillo A. Vitale M. Corbi G.. (2016). Serum levels of acyl-carnitines along the continuum from Normal to Alzheimer’s dementia. PLoS One 11:e0155694. doi: 10.1371/journal.pone.0155694 27196316 PMC4873244

[ref14] Cunnane S. Nugent S. Roy M. Courchesne-Loyer A. Croteau E. Tremblay S.. (2011). Brain fuel metabolism, aging, and Alzheimer’s disease. Nutrition 27, 3–20. doi: 10.1016/j.nut.2010.07.021, PMID: 21035308 PMC3478067

[ref15] Eisenbaum M. Pearson A. Gratkowski A. Mouzon B. Mullan M. Crawford F.. (2021). Influence of traumatic brain injury on extracellular tau elimination at the blood–brain barrier. Fluids Barriers CNS. 18, 48–13. doi: 10.1186/s12987-021-00283-y, PMID: 34702292 PMC8549249

[ref16] Fennema D. Phillips I. R. Shephard E. A. (2016). Trimethylamine and trimethylamine N-oxide, a Flavin-containing monooxygenase 3 (FMO3)-mediated host-microbiome metabolic Axis implicated in health and disease. Drug Metab Dispos. 44, 1839–1850. doi: 10.1124/dmd.116.070615 27190056 PMC5074467

[ref17] Gao X. Randell E. Zhou H. Sun G. (2018). Higher serum choline and betaine levels are associated with better body composition in male but not female population. PLoS One 13:e0193114. doi: 10.1371/journal.pone.0193114 29462191 PMC5819804

[ref18] Gawrys-Kopczynska M. Konop M. Maksymiuk K. Kraszewska K. Derzsi L. Sozanski K.. (2020). TMAO, a seafood-derived molecule, produces diuresis and reduces mortality in heart failure rats. eLife 9:e57028. doi: 10.7554/eLife.57028 32510330 PMC7334024

[ref19] Goldstein G. W. (1979). Relation of potassium transport to oxidative metabolism in isolated brain capillaries. J. Physiol. 286, 185–195. doi: 10.1113/jphysiol.1979.sp012613, PMID: 439024 PMC1281565

[ref20] González-Domínguez R. García-Barrera T. Gómez-Ariza J. L. (2014). Metabolomic study of lipids in serum for biomarker discovery in Alzheimer’s disease using direct infusion mass spectrometry. J. Pharm. Biomed. Anal. 98, 321–326. doi: 10.1016/j.jpba.2014.05.023 24992214

[ref21] Hingorani V. Brecher P. (1987). Glucose and fatty acid metabolism in normal and diabetic rabbit cerebral microvessels. Am. J. Physiol. Metab. 252, E648–E653. doi: 10.1152/ajpendo.1987.252.5.E648, PMID: 3578513

[ref22] Horgusluoglu E. Neff R. Song W. Wang M. Wang Q. Arnold M.. (2021). Integrative metabolomics-genomics approach reveals key metabolic pathways and regulators of Alzheimer’s disease. Alzheimers Dement 18:1–19. doi: 10.1002/alz.12468 34757660 PMC9085975

[ref23] Hoyles L. Pontifex M. G. Rodriguez-Ramiro I. Anis-Alavi M. A. Jelane K. S. Snelling T.. (2021). Regulation of blood–brain barrier integrity by microbiome-associated methylamines and cognition by trimethylamine N-oxide. Microbiome 9:235. doi: 10.1186/s40168-021-01181-z, PMID: 34836554 PMC8626999

[ref24] Huguenard C. J. C. Cseresznye A. Evans J. E. Darcey T. Nkiliza A. Keegan A. P.. (2022). APOE ε4 and Alzheimer’s disease diagnosis associated differences in L-carnitine, GBB, TMAO and acylcarnitines in blood and brain. Curr. Res. Transl. Med. 71:103362. doi: 10.1016/j.retram.2022.103362 36436355 PMC10066735

[ref25] Huguenard C. J. C. Cseresznye A. Evans J. E. Oberlin S. Langlois H. Ferguson S.. (2020). Plasma Lipidomic analyses in cohorts with mTBI and/or PTSD reveal lipids differentially associated with diagnosis and APOE ε4 carrier status. Front. Physiol. 11:12. doi: 10.3389/fphys.2020.00012 32082186 PMC7005602

[ref26] Huo Z. Yu L. Yang J. Zhu Y. Bennett D. A. Zhao J. (2020). Brain and blood metabolome for Alzheimer’s dementia: findings from a targeted metabolomics analysis. Neurobiol. Aging 1, 123–133. doi: 10.1016/j.neurobiolaging.2019.10.014 PMC6995427 31785839

[ref27] Jarrell Z. R. Smith M. R. Hu X. Orr M. Liu K. H. Quyyumi A. A.. (2020). Plasma acylcarnitine levels increase with healthy aging. Aging (Albany NY). 12, 13555–13570. doi: 10.18632/aging.103462 32554854 PMC7377890

[ref28] Jethva P. N. Udgaonkar J. B. (2018). The Osmolyte TMAO modulates protein folding cooperativity by altering global protein stability. Biochem. Int. 57, 5851–5863. doi: 10.1021/acs.biochem.8b00698, PMID: 30199620

[ref29] Johnson L. A. Torres E. R. Weber Boutros S. Patel E. Akinyeke T. Alkayed N. J.. (2019). Apolipoprotein E4 mediates insulin resistance-associated cerebrovascular dysfunction and the post-prandial response. J. Cereb. Blood Flow Metab. 39, 770–781. doi: 10.1177/0271678X17746186, PMID: 29215310 PMC6498752

[ref30] Jones L. L. McDonald D. A. Borum P. R. (2010). Acylcarnitines: role in brain. Prog. Lipid. Res. 49, 61–75. doi: 10.1016/j.plipres.2009.08.004 19720082

[ref31] Koeth R. Culley M. Wang Z. Nemet I. Kirsop J. Org E.. (2019). Crotonobetaine is a Proatherogenic gut microbiota metabolite of L-carnitine. J. Am. Coll. Cardiol. 73:14. doi: 10.1016/S0735-1097(19)30623-0

[ref32] Koeth R. A. Lam-Galvez B. R. Kirsop J. Wang Z. Levison B. S. Gu X.. (2019). L-carnitine in omnivorous diets induces an atherogenic gut microbial pathway in humans. J. Clin. Invest. 129, 373–387. doi: 10.1172/JCI94601, PMID: 30530985 PMC6307959

[ref33] Koeth R. A. Levison B. S. Culley M. K. Buffa J. A. Wang Z. Gregory J. C.. (2014). γ-Butyrobetaine is a proatherogenic intermediate in gut microbial metabolism of L-carnitine to TMAO. Cell Metab. 20, 799–812. doi: 10.1016/j.cmet.2014.10.006, PMID: 25440057 PMC4255476

[ref34] Koeth R. A. Wang Z. Levison B. S. Buffa J. A. Org E. Sheehy B. T.. (2013). Intestinal microbiota metabolism of l-carnitine, a nutrient in red meat, promotes atherosclerosis. Nat Med. 19, 576–585. doi: 10.1038/nm.3145, PMID: 23563705 PMC3650111

[ref35] Kristoffer B. Skoog I. Gustafson D. R. (2015). 37 years of body mass index and dementia: effect modification by the APOE genotype: observations from the prospective population study of women in Gothenburg, Sweden. J. Alzheimers Dis. 48, 1119–1127. doi: 10.3233/JAD-150326 26402098

[ref36] Lemaitre R. N. Jensen P. N. Wang Z. Fretts A. M. McKnight B. Nemet I.. (2021). Association of Trimethylamine N-oxide and related metabolites in plasma and incident type 2 diabetes: the cardiovascular health study. JAMA Netw. Open 4, 1–15. doi: 10.1001/jamanetworkopen.2021.22844 PMC8397925 34448864

[ref37] Lever M. Atkinson W. George P. M. Chambers S. T. (2007). Sex differences in the control of plasma concentrations and urinary excretion of glycine betaine in patients attending a lipid disorders clinic. Clin. Biochem. 40, 1225–1231. doi: 10.1016/j.clinbiochem.2007.05.021, PMID: 17706956

[ref38] Lever M. Sizeland P. C. B. Bason L. M. Hayman C. M. Chambers S. T. (1994). Glycine betaine and proline betaine in human blood and urine. BBA – Gen Subj. 1200, 259–264. doi: 10.1016/0304-4165(94)90165-1, PMID: 8068711

[ref39] Lim W. L. F. Huynh K. Chatterjee P. Martins I. Jayawardana K. S. Giles C.. (2020). Relationships between plasma lipids species, gender, risk factors, and Alzheimer’s disease. J. Alzheimers Dis. 76, 303–315. doi: 10.3233/JAD-191304, PMID: 32474467 PMC7369125

[ref40] Lin C. N. Huang C. C. Huang K. L. Lin K. J. Yen T. C. Kuo H. C. (2019). A metabolomic approach to identifying biomarkers in blood of Alzheimer’s disease. Ann. Clin. Transl. Neurol. 6, 537–545. doi: 10.1002/acn3.726 30911577 PMC6414491

[ref41] Longo N. Frigeni M. Pasquali M. (2016). Carnitine transport and fatty acid oxidation. Biochim. Biophys. Acta, Mol. Cell Res. 1863, 2422–2435. doi: 10.1016/j.bbamcr.2016.01.023 PMC4967041 26828774

[ref42] Luciani M. Mueller D. Vanetta C. Diteepeng T. Von Eckardstein A. Aeschbacher S.. (2021). Trimethylamine-N-oxide (TMAO) is associated with cardiovascular mortality and vascular brain lesions in patients with atrial fibrillation. Eur. Heart J. 42:2021. doi: 10.1093/eurheartj/ehab724.0475/6394642 36593094

[ref43] Makar T. K. Cooper A. J. L. Tofel-Grehl B. Thaler H. T. Blass J. P. (1995). Carnitine, carnitine acetyltransferase, and glutathione in Alzheimer brain. Neurochem. Res. 20, 705–711. doi: 10.1007/BF01705539, PMID: 7566367

[ref44] Makowski L. Noland R. C. Koves T. R. Xing W. Ilkayeva O. R. Muehlbauer M. J.. (2009). Metabolic profiling of PPARα −/− mice reveals defects in carnitine and amino acid homeostasis that are partially reversed by oral carnitine supplementation. FASEB J. 23, 586–604. doi: 10.1096/fj.08-119420, PMID: 18945875 PMC2630792

[ref45] Mapstone M. Cheema A. K. Fiandaca M. S. Zhong X. Mhyre T. R. Macarthur L. H.. (2014). Plasma phospholipids identify antecedent memory impairment in older adults. Nat. Med. 20, 415–418. doi: 10.1038/nm.3466, PMID: 24608097 PMC5360460

[ref46] Masters C. L. Bateman R. Blennow K. Rowe C. C. Sperling R. A. Cummings J. L. (2015). Alzheimer’s disease. Nat. Rev. Dis. Prim. 1, 1–18. doi: 10.1038/nrdp.2015.56 27188934

[ref47] Meadows J. A. Wargo M. J. (2015). Carnitine in bacterial physiology and metabolism. Microbiol (United Kingdom). 161, 1161–1174. doi: 10.1099/mic.0.000080 PMC4635513 25787873

[ref48] Nho K. Kueider-Paisley A. Arnold M. Mahmoudiandehkordi S. Risacher S. L. Louie G.. (2022). Serum metabolites associated with brain amyloid beta deposition, cognition and dementia progression. Brain Commun. 3:fcab139. doi: 10.1093/braincomms/fcab139 PMC8361396 34396103

[ref49] Ojo J. O. Reed J. M. Crynen G. Vallabhaneni P. Evans J. Shackleton B.. (2021). APOE genotype dependent molecular abnormalities in the cerebrovasculature of Alzheimer’s disease and age-matched non-demented brains. Mol Brain. 14, 110–124. doi: 10.1186/s13041-021-00803-9, PMID: 34238312 PMC8268468

[ref50] Oldendorf W. H. Brown W. J. (1975). Greater number of capillary endothelial cell mitochondria in brain than in muscle. Proc. Soc. Exp. Biol. Med. 149, 736–738. doi: 10.3181/00379727-149-38889, PMID: 1144461

[ref51] Qi G. Mi Y. Shi X. Gu H. Brinton R. D. Yin F. (2021). ApoE4 impairs neuron-astrocyte coupling of fatty acid metabolism. Cell Rep. 34:108572. doi: 10.1016/j.celrep.2020.108572, PMID: 33406436 PMC7837265

[ref52] Ramsay R. R. Gandour R. D. Van Der Leij F. R. (2001). Molecular enzymology of carnitine transfer and transport. Biochim. Biophys. Acta 1546, 1546, 21–43. doi: 10.1016/S0167-4838(01)00147-9 11257506

[ref53] Reiman E. M. Caselli R. J. Yun L. S. Chen K. Bandy D. Minoshima S.. (1996). Preclinical evidence of Alzheimer’s disease in persons homozygous for the ε4 allele for apolipoprotein E. N. Engl. J. Med. 334, 752–758. doi: 10.1056/NEJM199603213341202 8592548

[ref54] Reiman E. M. Chen K. Alexander G. E. Caselli R. J. Bandy D. Osborne D.. (2005). From the cover: correlations between apolipoprotein E 4 gene dose and brain-imaging measurements of regional hypometabolism. Proc. Natl. Acad. Sci. U. S. A. 102, 8299–8302. doi: 10.1073/pnas.0500579102, PMID: 15932949 PMC1149416

[ref55] Reuter S. E. Evans A. M. Chace D. H. Fornasini G. (2008). Determination of the reference range of endogenous plasma carnitines in healthy adults. Ann. Clin. Biochem. 45, 585–592. doi: 10.1258/acb.2008.008045, PMID: 18782814

[ref56] Rinaldo P. Cowan T. M. Matern D. (2008). Acylcarnitine profile analysis. Genet Med. 10, 151–156. doi: 10.1097/GIM.0b013e3181614289 18281923

[ref57] Schooneman M. G. Vaz F. M. Houten S. M. Soeters M. R. (2013). Acylcarnitines: reflecting or inflicting insulin resistance? Diabetes 62, 1–8. doi: 10.2337/db12-0466, PMID: 23258903 PMC3526046

[ref58] Shang Y. Mishra A. Wang T. Wang Y. Desai M. Chen S.. (2020). Evidence in support of chromosomal sex influencing plasma based metabolome vs APOE genotype influencing brain metabolome profile in humanized APOE male and female mice. PLoS One 15, e0225392–e0225321. doi: 10.1371/journal.pone.0225392, PMID: 31917799 PMC6952084

[ref59] Shinohara M. Kanekiyo T. Tachibana M. Kurti A. Shinohara M. Fu Y.. (2020). Apoe2 is associated with longevity independent of alzheimer’s disease. eLife 9, 1–16. doi: 10.7554/eLife.62199 PMC7588231 33074098

[ref60] Steinberg D. Parthasarathy S. Carew T. Khoo J. Witztum J. (1977). Cerebral edema: role of fatty acid metabolism of brain capillaries. N. Engl. J. Med. 296, 632–633. doi: 10.1056/NEJM197703172961115, PMID: 840248

[ref61] Sullivan P. M. Mezdour H. Aratani Y. Knouff C. Najib J. Reddick R. L.. (1997). Targeted replacement of the mouse apolipoprotein E gene with the common human APOE3 allele enhances diet-induced hypercholesterolemia and atherosclerosis *. J. Biol. Chem. 272, 17972–17980. doi: 10.1074/jbc.272.29.17972, PMID: 9218423

[ref62] Toledo J. B. Arnold M. Kastenmüuller G. Chang R. Baillie R. A. Han X.. (2017). Metabolic network failures in Alzheimer’s disease: a biochemical road map. Alzheimers Dement 13:965. doi: 10.1016/J.JALZ.2017.01.020 28341160 PMC5866045

[ref63] Tran T. T. T. Corsini S. Kellingray L. Hegarty C. Le G. G. Narbad A.. (2019). APOE genotype influences the gut microbiome structure and function in humans and mice: relevance for Alzheimer’s disease pathophysiology. FASEB J. 33, 8221–8231. doi: 10.1096/fj.201900071R, PMID: 30958695 PMC6593891

[ref64] Van Der Velpen V. Teav T. Gallart-Ayala H. Mehl F. Konz I. Clark C.. (2019). Systemic and central nervous system metabolic alterations in Alzheimer’s disease. Alzheimers Res. Ther. 11, 1–12. doi: 10.1186/s13195-019-0551-7 31779690 PMC6883620

[ref65] Varma V. R. Oommen A. M. Varma S. Casanova R. An Y. Andrews R. M.. (2018). Brain and blood metabolite signatures of pathology and progression in Alzheimer disease: a targeted metabolomics study. PLoS Med. 15:e1002482. doi: 10.1371/journal.pmed.1002482, PMID: 29370177 PMC5784884

[ref66] Veitch D. P. Weiner M. W. Aisen P. S. Beckett L. A. Cairns N. J. Green R. C.. (2019). Understanding disease progression and improving Alzheimer’s disease clinical trials: recent highlights from the Alzheimer’s Disease Neuroimaging Initiative. Alzheimer’s Dement 15, 106–152. doi: 10.1016/j.jalz.2018.08.005, PMID: 30321505

[ref67] Vogt N. M. Romano K. A. Darst B. F. Engelman C. D. Johnson S. C. Carlsson C. M.. (2018). The gut microbiota-derived metabolite trimethylamine N-oxide is elevated in Alzheimer’s disease. Alzheimer’s Res. Ther. 10, 1–8. doi: 10.1186/s13195-018-0451-2, PMID: 30579367 PMC6303862

[ref68] Wanders R. J. A. Waterham H. R. Ferdinandusse S. (2016). Metabolic interplay between peroxisomes and other subcellular organelles including mitochondria and the endoplasmic reticulum. Front. Cell Dev. Biol. 3:83. doi: 10.3389/fcell.2015.00083 26858947 PMC4729952

[ref69] Wang Z. Klipfell E. Bennett B. J. Koeth R. Levison B. S. Dugar B.. (2011). Gut flora metabolism of phosphatidylcholine promotes cardiovascular disease. Nature. 472, 57–63. doi: 10.1038/nature09922, PMID: 21475195 PMC3086762

[ref70] Xia Y. Li Q. Zhong W. Dong J. Wang Z. Wang C. (2011). L-carnitine ameliorated fatty liver in high-calorie diet/STZ-induced type 2 diabetic mice by improving mitochondrial function. Diabetol. Metab. Syndr. 3, 1–10. doi: 10.1186/1758-5996-3-31 22082204 PMC3226540

[ref71] Yao J. Rettberg J. R. Klosinski L. P. Cadenas E. Brinton R. D. (2011). Shift in brain metabolism in late onset Alzheimer’s disease: implications for biomarkers and therapeutic interventions. Mol. Aspects Med. 32, 247–257. doi: 10.1016/j.mam.2011.10.005, PMID: 22024249 PMC3658304

[ref72] Yassine H. N. Croteau E. Rawat V. Hibbeln J. R. Rapoport S. I. Cunnane S. C.. (2017). DHA brain uptake and APOE4 status: a PET study with [1-11C]-DHA. Alzheimers Res. Ther. 9:23. doi: 10.1186/s13195-017-0250-1, PMID: 28335828 PMC5364667

[ref73] Yassine H. N. Finch C. E. (2020). APOE alleles and diet in brain aging and Alzheimer’s disease. Front. Aging Neurosci. 12:150. doi: 10.3389/fnagi.2020.00150 32587511 PMC7297981

[ref74] Zhao N. Liu C. C. Van Ingelgom A. J. Martens Y. A. Linares C. Knight J. A.. (2017). Apolipoprotein E4 impairs neuronal insulin signaling by trapping insulin receptor in the endosomes. Neuron. 96, 115.e5–129.e5. doi: 10.1016/j.neuron.2017.09.003, PMID: 28957663 PMC5621659

