# Supplementary material for: Age and APOE affect L-carnitine system metabolites in the brain in the APOE-TR model
Source: Front Aging Neurosci. 2023 Jan 6;14:1059017. doi: 10.3389/fnagi.2022.1059017 (PMC9853982; doi:10.3389/fnagi.2022.1059017)
Supplement: Supplementary file 1 [file Data_Sheet_1.docx]

|  |  | **APOE-TR mice**  ***n* = 50** | | | | | | | | | | |
| --- | --- | --- | --- | --- | --- | --- | --- | --- | --- | --- | --- | --- |
| **Timepoint** |  | **10 weeks** | | | | **25 weeks** | | | | **50 weeks** | | |
| ***APOE* genotype** |  | **E2/E2** | **E3/E3** | **E4/E4** | **E2/E2** | | **E3/E3** | **E4/E4** | **E2/E2** | | **E3/E3** | **E4/E4** |
| **Numbers** |  | *n* = 6 | *n* = 6 | *n* = 6 | *n* = 5 | | *n* = 5 | *n* = 6 | *n* = 6 | | *n* = 6 | *n* = 4 |
| **Age in weeks** (average ± SD) |  | 10 ± 0 | 10 ± 0 | 10 ± 0 | 25 ± 0 | | 25 ± 0 | 25 ± 0 | 49 ± 2 | | 50 ± 0 | 50 ± 0 |
| **Sex** (*n)* | Male | 3 | 3 | 3 | 2 | | 3 | 3 | 3 | | 3 | 3 |
|  | Female | 3 | 3 | 3 | 3 | | 2 | 3 | 3 | | 3 | 1 |

**Table 1S.** APOE-TR mice (timepoint study) summary characteristics.

**Table 2S.** APOE-TR mice (cerebrovascular uptake study) summary characteristics.

| **APOE-TR**  ***n* = 22** | | | | |
| --- | --- | --- | --- | --- |
| ***APOE* genotype** |  | **E2/E2** | **E3/E3** | **E4/E4** |
| **Numbers** |  | *n* = 6 | *n* = 8 | *n* = 8 |
| **Age in weeks** (average ± SD) |  | 26 ± 0 | 26 ± 0 | 26 ± 0 |
| **Weight in grams**  (average ± SD) |  | 30 ± 6 | 32 ± 7 | 30 ± 6 |
| **Sex** (*n)* | Male | 2 | 4 | 4 |
|  | Female | 4 | 4 | 4 |

**Table 3S.** APOE-TR mice (L-carnitine challenge study) summary characteristics. Abbreviations; L-car: L-carnitine.

| **APOE-TR**  ***n* = 47** | | | | | | | |
| --- | --- | --- | --- | --- | --- | --- | --- |
| **Challenge** |  | **Vehicle** | **L-car** | **Vehicle** | **L-car** | **Vehicle** | **L-car** |
| ***APOE* genotype** |  | **E2/E2** | **E2/E2** | **E3/E3** | **E3/E3** | **E4/E4** | **E4/E4** |
| **Numbers** |  | *n* = 8 | *n* = 8 | *n* = 8 | *n* = 7 | *n* = 8 | *n* = 8 |
| **Age in weeks** (average ± SD) |  | 57 ± 1 | 57 ± 1 | 53 ± 1 | 53 ± 1 | 49 ± 4 | 49 ± 4 |
| **Weight in grams**  (average ± SD) |  | 40 ± 7 | 36 ± 9 | 39 ± 6 | 39 ± 6 | 31 ± 6 | 29 ± 5 |
| **Sex** (*n)* | Male | 4 | 4 | 4 | 3 | 4 | 4 |
|  | Female | 4 | 4 | 4 | 4 | 4 | 4 |

**Table 4S**. Acylcarnitine assay inclusion list. Abbreviations; Cx:y-CAR: acylcarnitines, Cx:y-OH-CAR: hydroxy acylcarnitines, Cx:y-DC-CAR: dicarboxy acylcarnitines, GBB: γ-butyrobetaine, IS: Internal Standard, N/A: not applicable, TMAO: trimethylamine-n-oxide.

| Name | Formula | Parent [*m/z*] | Daughter [*m/z*] | Type | Normalizing IS |
| --- | --- | --- | --- | --- | --- |
| d9 TMAO | C3D9NO | 85.1327 | 66.116 | IS | N/A |
| d9 L-carnitine | C7H6D9NO3 | 171.166 | 85.029 | IS | N/A |
| d3 C2:0 | C9H14D3NO4 | 207.142 | 85.029 | IS | N/A |
| d3 C3:0-CAR | C10H16D3NO4 | 221.158 | 85.029 | IS | N/A |
| d3 C4:0-CAR | C11H18D3NO4 | 235.174 | 85.029 | IS | N/A |
| d9 C5:0-CAR | C12H14D9NO4 | 255.227 | 85.029 | IS | N/A |
| d3 C5:0-OH-CAR | C12D3H20NO5 | 265.1843 | 85.029 | IS | N/A |
| d3 C5:0-DC-CAR | C12D3H18NO6 | 279.1635 | 85.029 | IS | N/A |
| d3 C8:0-CAR | C15H26D3NO4 | 291.236 | 85.029 | IS | N/A |
| d9 C12:0-CAR | C19D9H28NO4 | 353.3366 | 85.029 | IS | N/A |
| d9 C14:0-CAR | C21H32D9NO4 | 381.368 | 85.029 | IS | N/A |
| d3 C16:0-CAR | C23H42D3NO4 | 403.362 | 85.029 | IS | N/A |
| d3 C16:0-OH-CAR | C23D3H42NO5 | 419.3564 | 85.029 | IS | N/A |
| d3 C18:0-CAR | C25D3H46NO4 | 431.3928 | 85.029 | IS | N/A |
| TMAO | C3H9NO | 76.076 | 58.066 | Target | d9 TMAO |
| GBB | C7H16NO2 | 146.118 | 87.045 | Target | d9 L-carnitine |
| L-carnitine | C7H15NO3 | 162.113 | 85.029 | Target | d9 L-carnitine |
| C2:0-CAR | C9H17NO4 | 204.123 | 85.029 | Target | d3 C2:0-CAR |
| C3:0-CAR | C10H19NO4 | 218.139 | 85.029 | Target | d3 C3:0-CAR |
| C4:0-CAR* | C11H21NO4 | 232.154 | 85.029 | Target | d3 C4:0-CAR |
| C5:0-CAR* | C12H23NO4 | 246.171 | 85.029 | Target | d9 C5:0-CAR |
| C5-OH-CAR | C12H23NO5 | 262.165 | 85.029 | Target | d3 C5:0-OH-CAR |
| C5-DC-CAR | C12H21NO6 | 276.145 | 85.029 | Target | d3 C5:0-DC-CAR |
| C5:1-CAR | C12H21NO4 | 244.154 | 85.029 | Target | d9 C5:0-CAR |
| C6:0-CAR | C13H25NO4 | 260.186 | 85.029 | Target | d3 C8:0-CAR |
| C8:0-CAR | C15H29NO4 | 288.217 | 85.029 | Target | d3 C8:0-CAR |
| C8:1-CAR | C15H27NO4 | 286.202 | 85.029 | Target | d3 C8:0-CAR |
| C10:0-CAR | C17H33NO4 | 316.249 | 85.029 | Target | d3 C8:0-CAR |
| C10:0-OH-CAR | C17H33NO5 | 332.243 | 85.029 | Target | d3 C16:0-OH-CAR |
| C10:1-CAR | C17H31NO4 | 314.2326 | 85.029 | Target | d3 C8:0-CAR |
| C12:0-CAR | C19H37NO4 | 344.2795 | 85.029 | Target | d9 C12:0-CAR |
| C14:0-CAR | C21H41NO4 | 372.311 | 85.029 | Target | d9 C14:0-CAR |
| C14:1-CAR | C21H39NO4 | 370.2952 | 85.029 | Target | d9 C14:0-CAR |
| C14:2-CAR | C21H37NO4 | 368.28 | 85.029 | Target | d9 C14:0-CAR |
| C15:0-CAR | C22H43NO4 | 386.3265 | 85.029 | Target | d9 C14:0-CAR |
| C16:0-CAR | C23H45NO4 | 400.342 | 85.029 | Target | d3 C16:0-CAR |
| C16-OH-CAR | C23H45NO5 | 416.337 | 85.029 | Target | d3 C16:0-OH-CAR |
| C16:1-CAR | C23H43NO4 | 398.327 | 85.029 | Target | d3 C16:0-CAR |
| C18:0-CAR | C25H49NO4 | 428.374 | 85.029 | Target | d3 C18:0-CAR |
| C18:1-CAR | C25H47NO4 | 426.358 | 85.029 | Target | d3 C18:0-CAR |
| C18:2-CAR | C25H45NO4 | 424.343 | 85.029 | Target | d3 C18:0-CAR |
| C18:2-OH-CAR | C25H45NO5 | 440.337 | 85.029 | Target | d3 C16:0-OH-CAR |
| C18:3-CAR | C25H43NO4 | 422.327 | 85.029 | Target | d3 C18:0-CAR |
| C20:0-CAR | C27H53NO4 | 456.405 | 85.029 | Target | d3 C18:0-CAR |
| C20:1-CAR | C27H51NO4 | 454.39 | 85.029 | Target | d3 C18:0-CAR |
| C20:3-CAR | C27H47NO4 | 450.358 | 85.029 | Target | d3 C18:0-CAR |
| C20:4-CAR | C27H45NO4 | 448.342 | 85.029 | Target | d3 C18:0-CAR |
| C22:0-CAR | C29H57NO4 | 484.437 | 85.029 | Target | d3 C18:0-CAR |
| C24:0-CAR | C31H61NO4 | 512.468 | 85.029 | Target | d3 C18:0-CAR |
| C26:0-CAR | C33H65NO4 | 540.499 | 85.029 | Target | d3 C18:0-CAR |

*includes both isomers.

**Table 5S**. TMA assay inclusion list. Abbreviations; GBB: γ-butyrobetaine, IS: Internal Standard, N/A: not applicable, TMA: trimethylamine, TMAO: trimethylamine-n-oxide, TML: trimethyl-l-lysine.

| Name | Formula | [*m/z*] | Type | Normalizing IS |
| --- | --- | --- | --- | --- |
| d9-TMAO | C3H1NOD9 | 85.132 | IS | N/A |
| d9-TMA derivative | C7H7NO2D9 | 155.174 | IS | N/A |
| d3-L-carnitine | C7H13NO3D3 | 165.131 | IS | N/A |
| d9-TML | C9H12N2O2D9 | 198.216 | IS | N/A |
| d9-crotonobetaine | C7H14D9NO2 | 153.158 | IS | N/A |
| TMAO | C3H9NO | 76.075 | Target | d9-TMAO |
| TMA derivative | C7H16NO2 | 146.117 | Target | d9-TMA derivative |
| Crotonobetaine | C7H13NO2 | 144.101 | Target | d9-crotonobetaine |
| GBB | C7H15NO2 | 146.117 | Target | d3-L-carnitine |
| Betaine | C5H12NO2 | 118.086 | Target | d9-crotonobetaine |
| Choline | C5H14NO | 104.106 | Target | d3-L-carnitine |
| L-carnitine | C7H16NO3 | 162.112 | Target | d3-L-carnitine |
| TML | C9H20N2O2 | 189.159 | Target | d9-TML |

**Table 6S**. Palmitic acid-DHA assay inclusion list. Abbreviations; Cx:y: free fatty acid, DHA: docosahexaenoic acid, IS: Internal Standard, N/A: not applicable, UC13-C16:0: uniformly C13-labeled palmitic acid.

| Name | Formula | [*m/z*] | Type | Normalizing IS |
| --- | --- | --- | --- | --- |
| d2-C16:0 | C16D2H30O2 | 257.245 | IS | N/A |
| d5-DHA | C22D5H27O2 | 332.264 | IS | N/A |
| UC13-C16:0 | 13C16H32O2 | 271.286 | Target | d2-C16:0 |
| DHA | C22H32O2 | 327.232 | Target | d5-DHA |

**Figure 1S. Analysis of global effects shows age and *APOE* effects dominate brain acylcarnitine profiles while sex and *APOE* effects dominate peripheral profiles.** Bar graphs showing the number of acylcarnitine species significantly affected by *APOE* genotype, age, and sex in the cerebrovasculature, parenchyma, plasma, and liver.

Statistics: multiple comparisons were followed by a B-H correction. Abbreviations; sig. species: significant species.


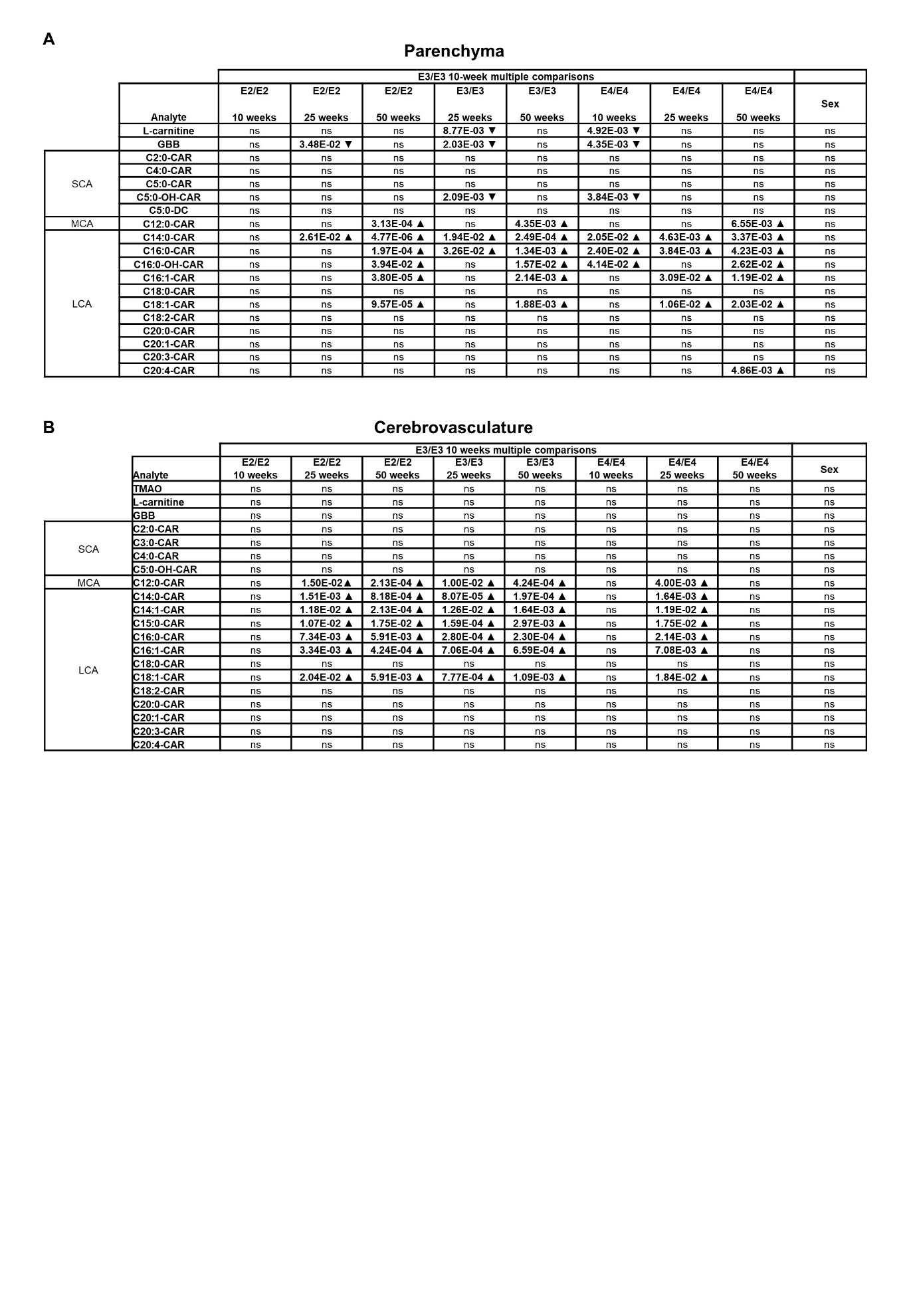


**Figure 2S. Multiple effects of age and *APOE* genotype on acylcarnitine specie in the brain. A** Table showing significant p-values for acylcarnitine specie in *APOE* genotype and age groups as well as sex groups in the brain parenchyma. **B** Table showing significant p-values for acylcarnitine specie in *APOE* genotype and age groups as well as sex groups in the cerebrovasculature.

Statistics: multiple comparisons with B-H correction. The “**▲**” symbol signifies an increase and “**▼**” a decrease compared to 10-week-old APOE3-TR mice. Numbers per group; 10-week APOE2-TR *n* = 6, 25-week APOE2-TR *n* = 5, 50-week APOE2-TR *n* = 6, 10-week APOE3-TR *n* = 6, 25-week APOE3-TR *n* = 5, 50-week APOE3-TR *n* = 6, 10-week APOE4-TR *n* = 6, 25-week APOE4-TR *n* = 6, 50-week APOE4-TR *n* = 4, males *n* = 26, females *n* = 24. Abbreviations; Cx:y-CAR: acylcarnitines, Cx:y-OH-CAR: hydroxy acylcarnitines, Cx:y-DC-CAR: dicarboxy acylcarnitines, GBB: γ-butyrobetaine, TMAO: trimethylamine-n-oxide.

**Figure 3S. *APOE* genotype and age affect parenchymal and cerebrovascular total acylcarnitine levels and APOE affects brain TMAO. A** Line graphs showing mean ± SD total brain parenchymal acylcarnitines in nM per gram of protein in *APOE* genotypes at different age timepoints. **B** Line graphs showing mean ± SD total brain parenchymal acylcarnitines in nM per gram of protein in APOE2-TR mice at different age timepoints, * comparison with 10 weeks, # comparison with 25 weeks. **C** Line graphs showing mean ± SD total brain parenchymal acylcarnitines in nM per gram of protein in APOE3-TR mice at different age timepoints, * comparison with 10 weeks. **D** Line graphs showing mean ± SD total brain parenchymal acylcarnitines in nM per gram of protein in APOE4-TR mice at different age timepoints. **E** Line graphs showing mean ± SD cerebrovascular total acylcarnitines in nM per gram of protein in different *APOE* genotypes and age timepoints. **F** Line graphs showing mean ± SD cerebrovascular total acylcarnitines in nM per gram of protein in APOE2-TR mice at different age timepoints, * comparison with 10 weeks. **G** Line graphs showing mean ± SD cerebrovascular total acylcarnitines in nM per gram of protein in APOE3-TR mice at different age timepoints, * comparison with 10 weeks. **H** Line graphs showing mean ± SD cerebrovascular total acylcarnitines in nM per gram of protein in APOE4-TR mice at different age timepoints, * comparison with 10 weeks. **I** Bar graphs showing all points with mean ± SD brain parenchymal TMAO in nM per gram of protein in different *APOE* genotypes.

Statistics: Multiple comparisons with B-H correction were performed on the data, *p<0.05, **p<0.01, ***p<0.001. Numbers per group; 10-week APOE2-TR *n* = 6, 25-week APOE2-TR *n* = 5, 50-week APOE2-TR *n* = 6, 10-week APOE3-TR *n* = 6, 25-week APOE3-TR *n* = 5, 50-week APOE3-TR *n* = 6, 10-week APOE4-TR *n* = 6, 25-week APOE4-TR *n* = 6, 50-week APOE4-TR *n* = 4, APOE2-TR *n* = 17, APOE3-TR *n* = 17, APOE4-TR *n* = 16. Abbreviation; TMAO: Trimethylamine-n-oxide.


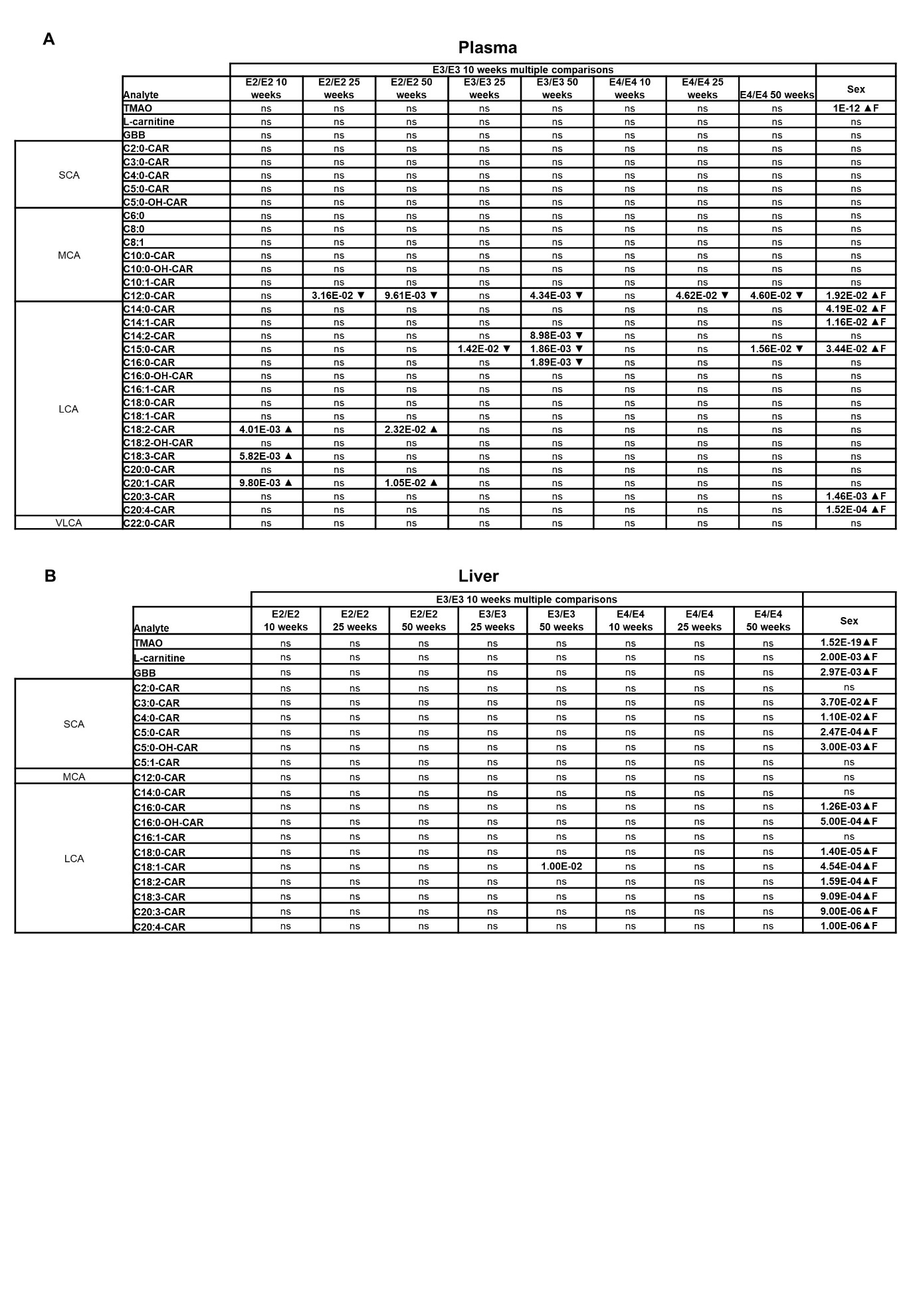


**Figure 4S. Multiple effects of age and *APOE* genotype on acylcarnitine specie in the plasma and liver. A** Table showing significant p-values for acylcarnitine specie in *APOE* genotype and age groups as well as sex groups in plasma. **B** Table showing significant p-values for acylcarnitine specie in *APOE* genotype and age groups as well as sex groups in the liver.

Statistics: multiple comparisons with B-H correction. The “**▲**” symbol signifies an increase and “**▼**” a decrease compared to 10-week-old APOE3-TR mice. The “**▲F**” symbol signifies an increase and “**▼F**” symbol a decrease in females compared to males. Numbers per group; 10-week APOE2-TR *n* = 6, 25-week APOE2-TR *n* = 5, 50-week APOE2-TR *n* = 6, 10-week APOE3-TR *n* = 6, 25-week APOE3-TR *n* = 5, 50-week APOE3-TR *n* = 6, 10-week APOE4-TR *n* = 6, 25-week APOE4-TR *n* = 6, 50-week APOE4-TR *n* = 4, males *n* = 26, females *n* = 24. Abbreviations; Cx:y-CAR: acylcarnitines, Cx:y-OH-CAR: hydroxylacylcarnitines, GBB: γ-butyrobetaine, TMAO: trimethylamine-n-oxide.

**Figure 5S. *APOE* affects plasma TMAO, SCA, and LCA levels but not total acylcarnitines. C** Line graphs showing mean ± SD plasma total acylcarnitines in nM with *APOE* genotypes at different age timepoints. **D** Line graphs showing mean ± SD plasma total acylcarnitines in nM in APOE2-TR mice at different age timepoints. **E** Line graphs showing mean ± SD plasma total acylcarnitines in nM in APOE3-TR mice at different age timepoints. **F** Line graphs showing mean ± SD plasma total acylcarnitines in nM in APOE4-TR mice at different age timepoints. **D** Bar graphs showing all points mean plasma TMAO ± SD in nM in different *APOE* genotypes in males. **E** Bar graphs showing all points mean plasma TMAO ± SD in μM in different *APOE* genotypes in females. **F** Bar graphs showing all points mean plasma SCA ± SD in nM in different *APOE* genotypes. **G** Bar graphs showing all points mean plasma LCA ± SD in nM in different *APOE* genotypes.

Statistics: multiple comparisons with B-H correction, *p<0.05, **p<0.01, ***p<0.001. Numbers per group; 10-week APOE2-TR *n* = 6, 25-week APOE2-TR *n* = 5, 50-week APOE2-TR *n* = 6, 10-week APOE3-TR *n* = 6, 25-week APOE3-TR *n* = 5, 50-week APOE3-TR *n* = 6, 10-week APOE4-TR *n* = 6, 25-week APOE4-TR *n* = 6, 50-week APOE4-TR *n* = 4, APOE2-TR (males) *n* = 8, APOE3-TR (males) *n* = 9, APOE4-TR (males) *n* = 9, APOE2-TR (females) *n* = 9, APOE3-TR (females) *n* = 8, APOE4-TR (females) *n* = 7, APOE2-TR *n* = 17, APOE3-TR *n* = 17, APOE4-TR *n* = 16. Abbreviations; LCA: long chain acylcarnitines, SCA: short chain acylcarnitines, TMAO: trimethylamine-n-oxide.

**Figure 6S. Sex and *APOE* genotype affect liver TMAO levels but not total acylcarnitines. A** Line graphs showing mean ± SD liver total acylcarnitines in nM per gram of protein with *APOE* genotypes at different age timepoints. **B** Line graphs showing mean ± SD liver total acylcarnitines in nM per gram of protein in APOE2-TR mice at different age timepoints. **C** Line graphs showing mean ± SD liver total acylcarnitines in nM per gram of protein in APOE3-TR mice at different age timepoints. **D** Line graphs showing mean ± SD liver total acylcarnitines in nM per gram of protein in APOE4-TR mice at different age timepoints. **E** Bar graphs showing all points mean liver TMAO ± SD in nM per gram of protein in different *APOE* genotypes in males. **F** Bar graphs showing all points mean liver TMAO ± SD in nM per gram of protein in different *APOE* genotypes in females.

Statistics: multiple comparisons with B-H correction, **p<0.01. Numbers per group: 10-week APOE2-TR *n* = 6, 25-week APOE2-TR *n* = 5, 50-week APOE2-TR *n* = 6, 10-week APOE3-TR *n* = 6, 25-week APOE3-TR *n* = 5, 50-week APOE3-TR *n* = 6, 10-week APOE4-TR *n* = 6, 25-week APOE4-TR *n* = 6, 50-week APOE4-TR *n* = 4, APOE2-TR (males) *n* = 8, APOE3-TR (males) *n* = 9, APOE4-TR (males) *n* = 9, APOE2-TR (females) *n* = 9, APOE3-TR (females) *n* = 8, APOE4-TR (females) *n* = 7. Abbreviation; TMAO: trimethylamine-n-oxide.

 **Figure 7S. No effect of *APOE* genotype on the cerebrovascular uptake of treatment compounds. A** Scatterplots with bar showing mean concentration of TMAO in nM per gram of protein ± SD in vehicle and compound treated cerebrovasculature. **B** Scatterplots with bar showing mean concentration of L-carnitine in nM per gram of protein ± SD in vehicle and compound treated cerebrovasculature. **C** Scatterplots with bar showing mean concentration of C6:0-CAR in nM per gram of protein ± SD in vehicle and compound treated cerebrovasculature. **D** Scatterplots with bar showing mean concentration of C12:0-CAR in nM per gram of protein ± SD in vehicle and compound treated cerebrovasculature. **E** Scatterplots with bar showing mean concentration of UC13-C16:0 in μM per gram of protein ± SD in vehicle and compound treated cerebrovasculature. **F** Scatterplots with bar showing mean concentration of DHA in μM per gram of protein ± SD in in vehicle and compound treated cerebrovasculature.

Statistics: related-sample analysis. Numbers per group (vehicle-treated sample pairs): E2 (vehicle) *n* = 6, E2 (treated) *n* = 6, E3 (vehicle) *n* = 8, E3 (treated) *n* = 8, E4 (vehicle) *n* = 8, E4 (treated) *n* = 8. Abbreviations; Cx:y-CAR: acylcarnitines, DHA: docosahexaenoic acid, TMAO: trimethylamine-n-oxide, UC13-C16:0: uniformly 13C-labelled palmitic acid.

**Figure 8S. *APOE* genotype modifies treatment-related changes in cerebrovascular acylcarnitines levels. A** Scatterplots with bar showing mean concentration of UC13-C16:0-CAR in nM per gram of protein ± SD in compound treated cerebrovasculature in different *APOE* genotypes. **B** Scatterplots with bar showing mean concentration of UC13-C2:0-CAR in nM per gram of protein ± SD in compound treated cerebrovasculature in different *APOE* genotypes. **C** Scatterplots with bar showing mean concentration of C15:0-CAR in nM per gram of protein ± SD in vehicle and compound treated cerebrovasculature in different *APOE* genotypes. **D** Scatterplots with bar showing mean concentration of C5:0-CAR in nM per gram of protein ± SD in vehicle and compound treated cerebrovasculature in different *APOE* genotypes. **E** Scatterplots with bar showing mean concentration of C3:0-CAR in nM per gram of protein ± SD in vehicle and compound treated cerebrovasculature in different *APOE* genotypes.

Statistics: related-sample analysis, within genotype vehicle-treated comparison, *p<0.05, **p<0.01, ***p<0.001. Numbers per group (vehicle-treated sample pairs): E2/E2 vehicle *n* = 6, E2/E2 treated *n* = 6, E3/E3 vehicle *n* = 8, E3/E3 treated *n* = 8, E4/E4 vehicle *n* = 8, E4/E4 treated *n* = 8. Abbreviations; Cx:y-CAR: acylcarnitines, FAO: fatty acid oxidation, UC13-C2:0-CAR: uniformly 13C labelled acetylcarnitine, UC13-C16:0: uniformly 13C labelled palmitic acid, UC13-C16:0-CAR: uniformly 13C labelled palmitoylcarnitine, UC13-C16:0-CoA: uniformly 13C labelled palmitoyl-CoA.

**Figure 9S. *APOE* genotype leads to differences in TMAO and ω-3/ω-6 acylcarnitine levels in the cerebrovasculature. A** Bar graphs showing all points with mean ± SD TMAO in nM per gram of protein in different *APOE* genotypes in vehicle treated cerebrovasculature. **B** Bar graphs showing all points with mean ± SD TMAO in nM per gram of protein by in different *APOE* genotypes in compound treated cerebrovasculature. **C** Scatterplots with bar showing mean concentration of C20:3-CAR in nM per gram of protein ± SD in the cerebrovasculature in different *APOE* genotypes. **D** Scatterplots with bar showing mean concentration of C20:4-CAR in nM per gram of protein ±SD in the cerebrovasculature in different *APOE* genotypes.

Statistics: multiple comparisons with B-H correction, *p<0.05, **p<0.01, ***p<0.001. Numbers per group: E2/E2 vehicle *n* = 6, E2/E2 treated *n* = 6, E3/E3 vehicle *n* = 8, E3/E3 treated *n* = 8, E4/E4 vehicle *n* = 8, E4/E4 treated *n* = 8, E2/E2 *n* = 12, E3/E3 *n* = 16, E4/E4 *n* = 16. Abbreviations; Cx:y-CAR: acylcarnitines, TMAO: trimethylamine-n-oxide.

**Figure 10S. No significant liver toxicity after 1-week oral L-carnitine challenge and significant effect of *APOE* on weight. A** Scatterplots with bar showing mean plasma concentration of AST in ng per ml ± SD in vehicle and L-carnitine challenged mice with sex and *APOE* genotype. **B** Scatterplots with bar showing mean body weight in gram ± SD with sex and *APOE* genotype.

Statistics: multiple comparisons with B-H correction, **p<0.01, ***p<0.001. Numbers per group: male E2/E2 (vehicle) *n* = 4, male E2/E2 (L-carnitine) *n* = 4, male E3/E3 (vehicle) *n* = 4, male E3/E3 (L-carnitine) *n* = 3, male E4/E4 (vehicle) *n* = 4, male E4/E4 (L-carnitine) *n* = 4 , female E2/E2 (vehicle) *n* = 4, female E2/E2 (L-carnitine) *n* = 4, female E3/E3 (vehicle) *n* = 4, female E3/E3 (L-carnitine) *n* = 4, female E4/E4 (vehicle) *n* = 4, female E4/E4 (L-carnitine) *n* = 4, E2/E2 (males) *n* = 8, E2/E2 (females) *n* = 8, E3/E3 (males) *n* = 7, E3/E3 (females) *n* = 8, E4/E4 (males) *n* = 8, E4/E4 (females) *n* = 8. Abbreviation; AST: aspartate aminotransferase.

**Figure 11S. Significant effects of L-carnitine challenge on liver and brain L-carnitine and brain crotonobetaine. A** Scatterplots with bar showing mean brain L-carnitine in nM per gram of protein ± SD in vehicle and L-carnitine challenged mice. **B** Scatterplots with bar showing mean brain concentration of crotonobetaine in nM per gram of protein ± SD in vehicle and L-carnitine challenged mice. **C** Scatterplots with bar showing mean liver concentration of L-carnitine in nM per gram of protein ± SD in vehicle and L-carnitine challenged mice.

Statistics: multiple comparisons with B-H correction, *p<0.05, ***p<0.001. Numbers per group: vehicle challenged *n* = 24, L-carnitine challenged *n* = 23.

| **L-carnitine challenge study** | | | | |
| --- | --- | --- | --- | --- |
|  |  | **Females vs Males** | | |
| **Assay** | **Analyte** | **Plasma** | **Brain** | **Liver** |
| **TMA** | TMAO | ↑↑↑ | ↑↑↑ | ↑↑↑ |
|  | TMA | ↓↓↓ | ↓↓↓ |  |
|  | Crotonobetaine |  |  |  |
|  | GBB |  |  | ↑↑ |
|  | Betaine | ↑↑↑ | ↑ | ↑↑ |
|  | Choline |  |  | ↓↓↓ |
|  | L-carnitine |  |  | ↑↑ |
|  | TML |  |  |  |
| **Acylcarnitine** | TMAO | ↑↑↑ | ↑↑↑ |  |
|  | GBB |  |  |  |
|  | L-carnitine |  |  |  |
|  | C2:0-CAR |  |  |  |
|  | C3:0-CAR | ↑↑ |  |  |
|  | C4:0-CAR |  |  |  |
|  | C5:0-CAR | ↑ |  |  |
|  | C5-OH-CAR |  |  |  |
|  | C5-DC-CAR | ↑ | ↑↑ |  |
|  | C5:1-CAR | ↑↑↑ |  |  |
|  | C8:0-CAR | ↓↓ |  |  |
|  | C8:1-CAR |  |  |  |
|  | C12:0-CAR | ↓↓ |  |  |
|  | C14:0-CAR | ↓↓ |  |  |
|  | C14:1-CAR | ↓↓↓ |  |  |
|  | C14:2-CAR |  | ↓ |  |
|  | C15:0-CAR |  |  |  |
|  | C16:0-CAR | ↓↓ |  |  |
|  | C16-OH-CAR | ↓↓↓ |  |  |
|  | C16:1-CAR | ↓↓↓ |  |  |
|  | C18:0-CAR |  |  |  |
|  | C18:1-CAR | ↓↓ |  |  |
|  | C18:2-CAR |  |  |  |
|  | C18:3-CAR |  |  |  |
|  | C20:0-CAR | ↓↓↓ |  |  |
|  | C20:1-CAR | ↓↓↓ | ↓ |  |
|  | C20:3-CAR |  |  |  |
|  | C20:4-CAR |  |  |  |
|  | C22:0-CAR |  |  |  |
|  | C24:0-CAR |  |  |  |

**Table 7S. Sex effects on L-carnitine gut-related metabolites and acylcarnitines are observed in the periphery and the brain regardless of challenge.** Table showing analytes significantly affected by sex in females compared to males.

Statistics: multiple comparisons with B-H correction. The arrows indicate the direction of change in females compared to males and the number of arrows corresponds to the statistical significance, ↓ or ↑: p<0.05, ↓↓ or ↑↑: p<0.01, and ↓↓↓ or ↑↑↑: p<0.001. Numbers per group: males *n* = 23, females *n* = 24. Abbreviations; Cx:y-CAR: acylcarnitines, Cx:y-OH-CAR: hydroxy acylcarnitines, Cx:y-DC-CAR: dicarboxy acylcarnitines, GBB: γ-butyrobetaine, TMA: trimethylamine, TMAO: trimethylamine-n-oxide.

**Liver**

**Brain**

**Plasma**

▲ L-carnitine

▲ TMAO

▲ Betaine

▲ Betaine

▲ Choline

▲ GBB

▲ TMA

▲ Crotonobetaine

▲ C15:0-CAR

**Figure 12S. Peripheral and brain L-carnitine metabolites are positively correlated.** Venn diagram depicting significant correlations between brain, liver, and plasma L-carnitine metabolites**.**

Statistics: Kendall’s tau-b with two-tailed significance threshold p<0.05, ▲: positive correlation. Abbreviations; Cx:y-CAR: acylcarnitines, GBB: γ-butyrobetaine,TMA: trimethylamine, TMAO: trimethylamine-n-oxide.
